# Patients with a Bicuspid Aortic Valve (BAV) Diagnosed with ECG-Gated Cardiac Multislice Computed Tomography—Analysis of the Reasons for Referral, Classification of Morphological Phenotypes, Co-Occurring Cardiovascular Abnormalities, and Coronary Artery Stenosis

**DOI:** 10.3390/jcm13133790

**Published:** 2024-06-27

**Authors:** Piotr Machowiec, Piotr Przybylski, Elżbieta Czekajska-Chehab, Andrzej Drop

**Affiliations:** Department of Radiology, Medical University of Lublin, 20-059 Lublin, Poland; dr.przybylski@gmail.com (P.P.); czekajska@gazeta.pl (E.C.-C.); andrzej.drop@umlub.pl (A.D.)

**Keywords:** bicuspid aortic valve, cardiac computed tomography, coronary stenosis

## Abstract

**Background/Objectives**: The aim of this study was to analyze a group of patients with a bicuspid aortic valve (BAV) examined with ECG-gated cardiac CT (ECG-CT), focusing on the assessment of the clinical reasons for cardiac CT, cardiovascular abnormalities coexisting with their BAV, and coronary artery stenosis. **Methods**: A detailed statistical analysis was conducted on 700 patients with a BAV from a group of 15,670 patients examined with ECG-CT. **Results**: The incidence of a BAV in ECG-CT was 4.6%. The most common reason for examination was suspicion of coronary heart disease—31.1%. Cardiovascular defects most frequently associated with a BAV were a VSD (4.3%) and coarctation of the aorta (3.6%), while among coronary anomalies, they were high-take-off coronary arteries (6.4%) and paracommissural orifice of coronary arteries (4.4%). The analysis of the coronary artery calcium index showed significantly lower values for type 2 BAV compared to other valve types (*p* < 0.001), with the lowest average age in this group of patients. Moreover, the presence of a raphe between the coronary and non-coronary cusps was associated with a higher rate of significant coronary stenosis compared to other types of BAVs (*p* < 0.001). **Conclusions**: The most common reason for referral for cardiac ECG-CT in the group ≤ 40-year-olds with a BAV was the suspicion of congenital cardiovascular defects, while in the group of over 40-year-olds, it was the suspicion of coronary artery disease. The incidence of cardiovascular abnormalities co-occurring with BAV and diagnosed with ECG-CT differs among specific patient subgroups. The presence of a raphe between the coronary and non-coronary cusps appears to be a potential risk factor for significant coronary stenosis in patients with BAVs.

## 1. Introduction

A bicuspid aortic valve (BAV) is the most common congenital heart defect, with a prevalence of 0.1–2% in the general population. A BAV is inherited as an autosomal dominant trait with incomplete penetrance and variable expression, due to its complex genetic architecture [1,2]. A BAV is associated with certain genetic syndromes like Turner syndrome, Loeys–Dietz syndrome, multisystemic smooth muscle dysfunction syndrome, and velocardiofacial syndrome, as well as complex congenital heart defects that affect the left ventricular outflow tract (LVOT) [3,4,5]. Although a BAV is linked with certain genetic syndromes and complex congenital heart defects, the majority of cases are isolated and non-syndromic. Most non-syndromic BAV cases cannot be explained by currently known single-gene mutations [1]. According to the evidence framework established by the Clinical Genome Resource for determining the pathogenicity of genetic variants, most of the genetic variants found in BAV patient cohorts do not meet these specific criteria [6]. There are also some other genetic syndromes with less frequent BAVs including Down syndrome, caused by trisomy of chromosome 21, Alagille syndrome, caused by mutation of the *NOTCH* ligands *JAG1* or *JAG2*, and Kabuki syndrome, caused by mutation of the epigenetic regulators *KMT2D* or *KDM6A* [1]. Due to the complex genetic model and significant genetic heterogeneity of BAV, only a few genes have been identified in association with the condition, leaving the majority of its genetic basis still unknown. Moreover, the role of environmental factors like longstanding abnormal blood flow and hypertension in BAV development is also considered [7].

The bicuspid aortic valve is often accompanied by additional congenital defects of the heart and other organs, which can determine the patient’s overall condition and affect their treatment progress. In most cases, those are associated with mutations within genes involved in embryogenesis of the aortic valve and other heart structures [8]. The most common defects of the heart and great vessels coexisting with a BAV include coarctation of the aorta, an atrial septal defect, a ventricular septal defect, and patent ductus arteriosus [9,10,11,12].

One of the first classifications of the bicuspid aortic valve was Roberts’ classification, which was based on autopsy material and specified two types of valves: R-L, where the right and left cusps are present, and A-P, where anterior and posterior cusps are visible [13]. Its further additions are the Brandenburg and Sabet classifications of BAVs [14,15]. The most widespread classification is that proposed by Sievers–Schmidtke, in which bicuspid aortic valves are divided into three main types, according to the number of raphes. The authors additionally distinguish several subtypes, based on the orientation of the raphes and cusps relative to the coronary sinuses [16]. Alluding to the International Consensus Classification and Nomenclature for the congenital bicuspid aortic valve condition, three types of bicuspid valves are recognized: the fused type (right–left cusp fusion, right-non-coronary cusp fusion, and left-non-coronary cusp fusion phenotypes); the two-sinus type (latero-lateral and antero-posterior phenotypes); and the partial-fusion type (forme fruste) [17]. Furthermore, the assessment of BAV symmetry for the fused BAV type was taken into account based on the angle between the commissures of the non-fused cusp. The International Consensus by Michelena et al. also distinguished two major forms of BAV aortopathy phenotypes—the ascending phenotype and the root phenotype [17].

Each morphological type is linked with distinct pathologies of the valve and the aorta and can even influence their prognosis [18]. Based on some studies, the AP morphotype (type 0 according to Sievers–Schmidtke) is related to having a larger annulus and a smaller ascending aorta [19]. It was reported that the higher prevalence of ascending aorta dilation was characteristic of a type 1 R-L BAV while aortic root dilation was more common in subjects with type 0 AP [20]. Moreover, the presence of a raphe (Type 1 and 2) was linked to a higher incidence of significant aortic stenosis and regurgitation [21]. Furthermore, a bicuspid aortic valve exerts different hemodynamics after TAVR based on the morphologic type. The type 1 BAV morphology demonstrates superior hemodynamics compared to a Sievers type 0 BAV [22]. It was hypothesized that various configurations of BAV, such as type 0 or type 1 R-L with fully fused cusps, might differently influence blood flow patterns in the aortic root and ascending aorta [23]. It was suggested that the various types of BAVs originate from separate embryological origins [24]. Little is known about embryological mechanisms responsible for the nonseparation of cusps in BAVs and even less for unicuspid aortic valve disease (type 2) [22]. Some individual studies suggest that 1 R-N BAVs stem from a morphogenetic defect occurring before outflow tract septation, whereas 1 R-L BAVs result from abnormal septation of the proximal part of the cardiac outflow tract [24].

Although a BAV can remain without clinical manifestations throughout a lifetime in many cases, in about one-third of the cases, a bicuspid aortic valve requires cardiac surgical intervention. Approximately 40% of them need isolated BAV repair, while about 60% undergo aortic replacement with reimplantation or remodeling [25]. The presence of a bicuspid aortic valve often leads to complications, such as aortic stenosis (in 59–81%, according to various studies) [15], aortic regurgitation (58–64% of patients with BAV) [26,27], co-occurrence of both, or aortic dilatation (10–35% of patients with a bicuspid aortic valve) [15,28,29]. In addition, the bicuspid aortic valve shows particular susceptibility to infective endocarditis, with the incidence of active inflammatory lesions in patients with a BAV oscillating at around 5% of the cases [30,31].

Echocardiography is considered the first-line imaging modality for the diagnosis of BAVs [32]. Transthoracic echocardiography (TTE) is often associated with the difficulty of obtaining an adequate acoustic window for imaging the bicuspid aortic valve [33]. Furthermore, the diagnostic sensitivity of transthoracic echocardiography may be limited in patients with large, extensive calcifications within the valve [34]. However, it is difficult to determine the presence/absence of a raphe or the position of the coronary artery ostium using TTE alone. TTE can identify the valvular phenotype in approximately 50% of patients, whereas 2D transesophageal echocardiography (TEE) works in 90.1% [35]. Due to its increased resolution and unobstructed visualization, TEE can provide more details related to the assessment of aortic valve morphology that are not obvious with TTE and can also facilitate direct planimetric measurements of the aortic orifice [36]. However, it is less accessible, more invasive, and may be associated with the risk of gastrointestinal disruption, esophageal bleeding, and the development of bacteremia [37]. A more accurate non-invasive test, i.e., ECG-gated multislice CT (MSCT), is very helpful in the assessment of a bicuspid aortic valve and can minimize artifacts caused by cardiac motion and improve image resolution [38]. Cardiac CT allows for accurate differentiation between tricuspid and bicuspid aortic valves, which can be important for preoperative planning. It additionally allows for simultaneous and comprehensive assessment of other cardiovascular structures, including evaluation of the coronary arteries. Furthermore, ECG gating becomes particularly useful in the diagnosis of valves with a residual raphe between the cusps, which typically require data acquisition during both systole and diastole of the heart [38,39].

In recent years, the group of indications for coronary computed tomography angiography (CCTA) has been expanding rapidly, particularly in diagnosing coronary artery disease. Based on the 2021 SCCT (Society of Cardiovascular Computed Tomography) report [40], CCTA is considered the first-line imaging in patients with stable angina without a history of coronary artery disease, for the assessment of coronary anomalies, and in patients with coronary artery bypass grafts, especially when the main aim is to assess their patency. It is also a recommended imaging option in patients with a history of coronary artery disease and concomitant stable angina, for the evaluation of coronary stents, and for the assessment of the coronary artery anatomy in patients with suspected aortic dissection. In addition, computed tomography angiography is considered an accurate modality for the assessment of acute and chronic thoracic aortic abnormalities (e.g., dissection or aneurysm) [41]. It is worth mentioning that the assessment of many cardiovascular structures, including extracardiac structures, takes place with other indications for ECG-gated cardiac CT.

There is extensive literature on the evaluation of bicuspid aortic valve complications with echocardiography, focusing mainly on aortic regurgitation and aortic stenosis [42,43,44,45,46]. Moreover, the association of the bicuspid aortic valve and aortopathy has been comprehensively described, both based on echocardiography [20,47,48,49,50] and computed tomography [51,52,53], and therefore an analysis of that problem was left out of this work. Our study aimed to assess the group structure of patients with a bicuspid aortic valve diagnosed or confirmed by ECG-CT using the Sievers–Schmidtke classification, to analyze the clinical reasons for referral for ECG-CT, to characterize the cardiovascular abnormalities coexisting with the bicuspid aortic valve, and to assess coronary artery stenosis, i.e., the aspects that have not been profoundly analyzed on larger study groups using CCTA data in any study published to date.

## 2. Materials and Methods

### 2.1. Study Design

In a group of 15,670 consecutive patients with ECG-gated CT performed for various reasons, 728 patients (4.6%) with a bicuspid aortic valve confirmed or diagnosed with ECG-gated cardiac CT in the period between 2008 and 2023 were eligible for this study.

**Figure 1:**
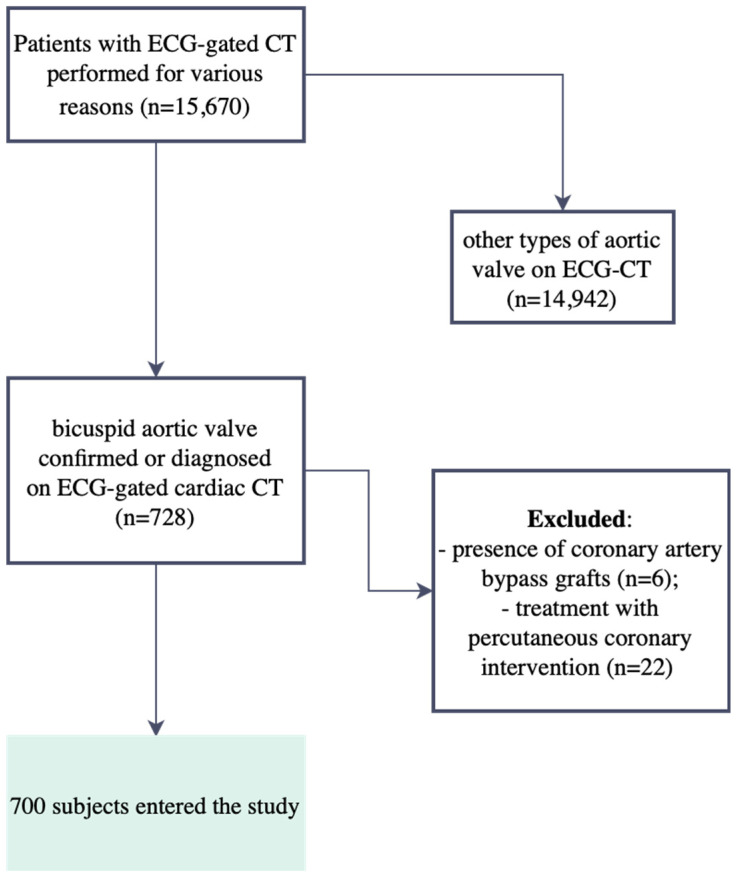
Study flowchart with inclusion and exclusion criteria.

To obtain a more homogeneous study group in terms of atherosclerotic lesions, patients with coronary artery bypass grafts (6 subjects—0.82%) and after percutaneous coronary intervention (22 subjects—3.0%) were excluded from further analysis. The final analyzed study group comprised 700 patients (Figure 1). The subsequent analysis included demographic factors, clinical factors, and CT-derived data. The patients’ ages were taken into account in numerically comparable age groups created by the authors: group I up to 40 years of age, group II between 41 and 60 years of age, and group III over 60 years of age. Patients diagnosed or confirmed by ECG-CT to have a bicuspid aortic valve were referred for this examination for various clinical reasons. The most common reason for referral was the diagnosis of coronary artery disease and follow-up after invasive treatment of coronary artery disease—about 30% of all cases.

### 2.2. Computed Tomography Protocol

The examinations were performed in the 1st Department of Radiology of the Medical University of Lublin, Poland, using a 256-slice Revolution CT scanner (General Electric Healthcare, Milwaukee, WI, USA)—49% of all examinations, and a 64-slice LightSpeed VCT (General Electric Healthcare, Milwaukee, WI, USA)—51% of all examinations. They were performed by means of native scanning with ECG gating to assess coronary artery calcification (calcium scoring) and after intravenous contrast agent administration. If the heart rate exceeded 65/min, a β-blocker was administered (usually metoprolol orally in a standard dose of 25–50 mg). In patients with suspected aortic dilatation or aortic valve disease, and in those investigated for complications related to cardiac pacemakers, the scan extent covered the area from the aortic arch to a level approximately 3 cm below the inferior margin of the heart. In all patients with suspected coarctation of the aorta in the precontrast phase, the scan range was extended to include the entire aorta. In the remaining cases, where the aorta was not dilated, typical native scanning was performed covering the area from 1–2 cm below the aortic bifurcation to 3 cm below the inferior margin of the heart. The technical parameters of coronary CTA (computed tomography angiography) for both scanners were as follows: for the 64-row scanner—collimation 64 × 0.6 mm, pitch 0.16:1–0.25:1, gantry rotation time 0.35 s; for the 256-row scanner—collimation 256 × 0.625 mm, gantry rotation time 0.28 s. In most subjects, a tube voltage of 120 kV was used, but in some patients, due to weight and age, the tube voltage was adjusted individually and was either 80, 100, or 140 kV. The amount of contrast agent in adults was 1 mL/kg (70–140 mL) on average, while in children, the dose was set individually according to weight, usually within the range of 1.5–2 mL/kg. The iodine contrasts used were either Ultravist 370, 370 mg I/mL (Bayer Pharma AG, Berlin, Germany) or Iomeron 400, 400 mg I/mL (Bracco Imaging Deutschland, Konstanz, Germany) administered at a flow rate of 4.5–6 mL/s. The scan delay was determined using the SmartPrep technique in the ascending aorta. In some clinical indications, it was individually decided to perform the second phase of scanning after 60 s. Subsequently, images were reconstructed in 10 series with a 10% R-R interval starting from phase 5% (5–95%). Once the reconstructed series were obtained, all data were transferred to one of the dedicated diagnostic consoles (Advantage Window 4.6 or 4.7 by GE) with cardiac CT evaluation software (CardIQ Xpress 2.0). Studies were performed in axial planes, multiplanar reconstructions (MPRs), maximum-intensity projections (MIPs), and volume reconstructions.

### 2.3. Analysis of Reasons for Referral for Cardiac CT in Patients with BAV

To analyze the reasons for referral for ECG-gated multislice computed tomography in a group of patients with a bicuspid aortic valve diagnosed or confirmed in this modality, 15 categories of reasons for referral were identified on the basis of clinical data reported by referring physicians: category 1—suspected coronary artery disease, category 2—suspicion or postoperative assessment of congenital cardiovascular defects excluding BAV, category 3—verification of a bicuspid aortic valve, 4—dilatated ascending aorta in echocardiography without initial diagnosis of a bicuspid aortic valve or aortic valve disease, 5—dilated ascending aorta in echocardiography with accompanying aortic valve disease (aortic stenosis, aortic insufficiency), or a bicuspid aortic valve, 6—suspicion of aortic dissection, 7—parametric assessment of acquired aortic valve disease (aortic stenosis, aortic insufficiency) without initial diagnosis of a bicuspid aortic valve, 8—parametric assessment of aortic stenosis or aortic insufficiency coexisting with a bicuspid aortic valve, 9—diagnosing cardiomyopathy causes, 10—diagnosing hypertension causes, 11—diagnosing arrhythmia, or suspicion of arrhythmogenic right ventricular dysplasia, 12—diagnosing complications related to pacemakers, 13—diagnosing abnormalities before planned ablation, 14—assessment of the heart and the aorta before transcatheter aortic valve implantation (TAVI), and 15—others, e.g., diagnosing pulmonary hypertension causes, suspicion of valvular vegetations, diagnosing dyspnea causes.

### 2.4. BAV Classification

**Figure 2:**
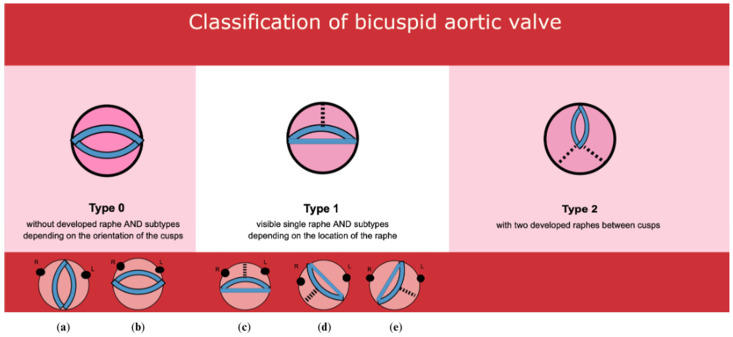
Classification of the bicuspid aortic valve based on Sievers H, Schmidtke C. A classification system for the bicuspid valve from 304 surgical specimens. J. Thorac. Cardiovasc. Surg. 2007, 133(5): 1226–1233—own sketch. (**a**) Type 0 A-P; (**b**) type 0 lateral; (**c**) type 1 R-L; (**d**) type 1 R-N; (**e**) type 1 L-N. The dashed line indicates the raphe between the cusps, while the black dots indicate the coronary arteries: R—right, L—left.

**Figure 3:**
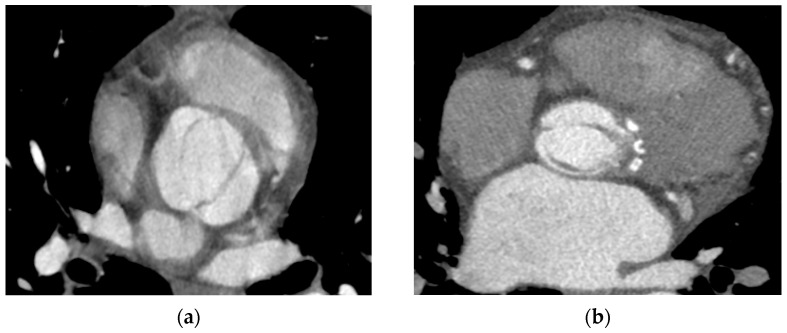

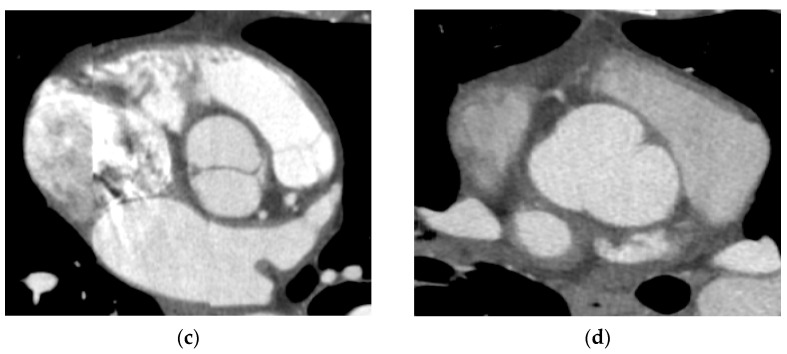
Type 0 bicuspid aortic valve (BAV), the so-called purely bicuspid aortic valve. (**a**,**c**) A-P valve with both coronary arteries departing from one sinus of Valsalva, (**b**,**d**) lateral valve with arteries departing from opposite sinuses. The top row shows open valves, and the bottom row shows closed valves.

**Figure 4:**
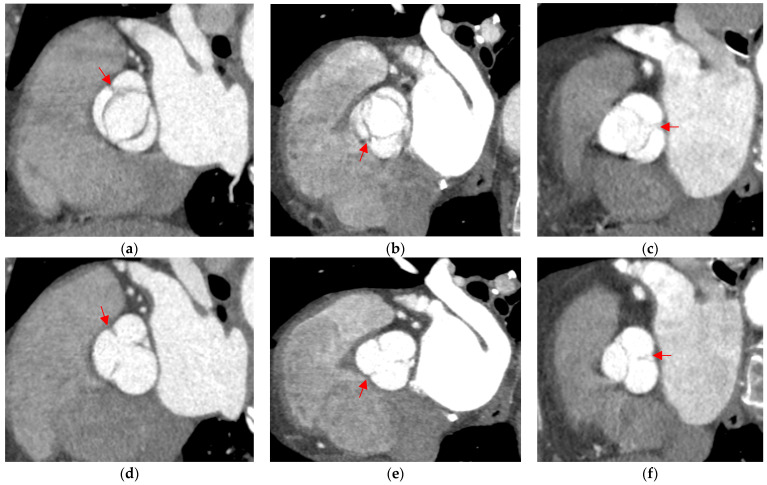
Type 1 BAV with raphe within fused cusps. (**a**,**d**) R-L valve with right and left cusps fused; (**b**,**e**) R-N valve with raphe between right and non-coronary cusps; (**c**,**f**) L-N valve with raphe between left and non-coronary cusps. The raphe on the dominant cusps is marked with a red arrow. The top row (**a**–**c**) shows open valves, and the bottom row (**d**,**e**) shows closed valves.

**Figure 5:**
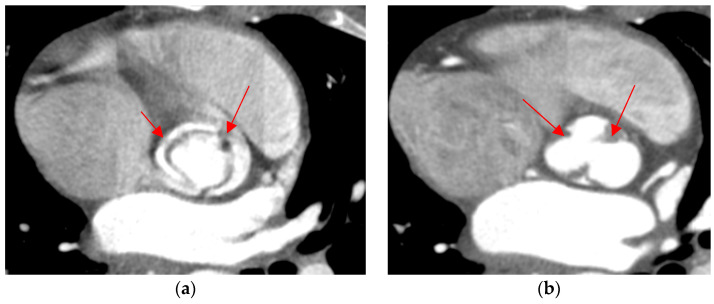
Type 2 BAV with two raphes. (**a**) Open valve; (**b**) closed valve. The raphes are marked with red arrows.

The department protocol for ECG-gated multislice cardiac computed tomography included the assessment of the great vessels, coronary arteries, and heart valves with parametric evaluation in view of their stenosis and regurgitation, as well as the determination of the morphological types of the valves and assessment of extracardiac lesions. The aortic valve was analyzed in all phases of the cardiac cycle, but the phase with its full opening (15%) and complete closure (75%) was usually selected for proper assessment. The BAV type was determined based on the Sievers–Schmidtke classification, in which type 0 was recognized by the presence of two cusps, two zones of parallel apposition, and two commissures. Type 1 was characterized by the additional presence of a single raphe on one of the cusps, while type 2 was diagnosed based on the presence of two raphes and only one zone of parallel apposition between the lobes. In addition, BAV types were divided into subtypes taking into account the orientation of the cusps (A-P and lateral) in the case of type 0 and the location of the raphe in type 1 (L-R, L-N, R-N) (Figure 2, Figure 3, Figure 4 and Figure 5). Moreover, we did not take the partial-fusion BAV into account during analysis, and it was treated as a variant imitating a bicuspid aortic valve. For this study, when characterizing the study group and assessing coronary artery stenosis, groups with subtype 1 R-N and subtype 1 L-N, i.e., subtypes with the presence of a raphe between the coronary and non-coronary cusps, were combined. In addition, due to the low prevalence of subtypes 0 A-P and 0 lateral, the type 0 bicuspid valve was not subdivided into subtypes when characterizing the study group and assessing coronary artery stenosis.

### 2.5. Classification of Cardiovascular Abnormalities Coexisting with BAV

Cardiovascular abnormalities detected in patients with a bicuspid aortic valve during ECG-gated multislice computed tomography were divided into three groups: group I—cardiovascular defects (G.I), group II—coronary anomalies (G.II), and group III—other cardiovascular abnormalities (G.III). Coronary anomalies were identified based on the Angelini (2007) classification [54]. This is the most accurate and comprehensive classification of coronary anomalies and the only one that describes coronary artery anomalies in detail, specifying low take-off and paracommissural orifice of the coronary arteries (A2b and A2c, according to Angelini). In our study, we included the two most important groups of coronary anomalies: anomalies of origin and anomalies of termination. In further analysis, category A2a (high take-off) and category A3b (origin from the ascending aorta) were combined into a common category of high-take-off coronary arteries. Cardiovascular defects and coronary anomalies (G.I and G.II) were treated as cardiovascular abnormalities of greater clinical significance. These groups were considered separately when assessing cardiovascular abnormalities co-occurring in patients with a bicuspid aortic valve, and to create a number of defects/person ratio enabling a comparison of the number of clinically significant cardiovascular abnormalities (G.I and G.II) per subject between the subgroups of the initial study group. Given the higher prevalence of left-dominant coronary circulation in patients with a BAV and other cardiovascular defects reported in the literature [55], we additionally assessed the percentage of left-dominant coronary circulation in patients with specific BAV types according to the Sievers–Schmidtke classification in male and female groups, as well as in age groups, of patients with a bicuspid aortic valve on CT.

### 2.6. Coronary Stenosis Severity Score

For the analysis of atherosclerotic lesions in the coronary arteries, the authors adopted a general classification, modeled on Coronary Artery Disease Reporting and Data System (CAD-RADS) 2.0, which takes into account the highest grade of coronary stenosis detected in any coronary artery. For the sake of further analysis, a common category 1 was created, which is a combination of CAD-RADS scores 1 and 2. The classification thus created was as follows:

Category 0 (corresponding to CAD-RADS 0)—no atherosclerotic lesions, stenosis 0%;
Category 1 (corresponding to CAD-RADS 1–2)—insignificant lesions, stenosis 1–49%;
Category 2 (corresponding to CAD-RADS 3)—less severe significant lesions, stenosis 50–69%;
Category 3 (corresponding to CAD-RADS 4)—significant lesions, stenosis 70–99%;
Category 4 (corresponding to CAD-RADS 5)—total coronary occlusion, 100% stenosis.

Coronary artery calcification was assessed using the Agatston index, and its value was then assigned to the appropriate P category, according to the CAD-RADS guidelines, defining the coronary plaque burden:

Calcium score 1–100 (P1 according to CAD-RADS)—mild amount of plaque;
Calcium score 101–300 (P2 according to CAD-RADS)—moderate amount of plaque;
Calcium score 301–999 (P3 according to CAD-RADS)—severe amount of plaque;
Calcium score > 1000 (P4 according to CAD-RADS)—extensive amount of plaque.

When interpreting the coronary index, percentiles were used to better represent the distribution of calcium scores across groups. They are a measure of the position in a statistical distribution that indicates what percentage of the data is below a given value. Five percentiles were determined—25th, 50th, 75th, and 90th (which are the main reference values in the Multi-Ethnic Study of Atherosclerosis (MESA)) [56], as well as an additional 95th percentile for the highest calcium scores.

During the analysis of coronary artery stenosis, demographic data—age, gender; clinical data—BMI, the presence of dyslipidemia, diabetes mellitus, atrial fibrillation, and hypertension; as well as CT-derived parameters of left ventricular function were taken into account. We also created an age- and gender-matched control group showing the burden of disease in patients with a tricuspid aortic valve. It was isolated from a group of 15,670 subjects with ECG-gated CT performed for various reasons. To ensure that matched subjects were comparable, we calculated the standardized mean difference. A value below 0.1 indicated that the variable was well-balanced between groups.

After analyzing the percentage of subjects with significant coronary artery stenosis in each BAV type, a univariate logistic regression was conducted, considering the BAV type characterized by the highest frequency of significant coronary stenosis as the risk factor for significant coronary stenosis. The result of the analysis is presented as an odds ratio (OR) together with a 95% confidence interval. The OR ratio determines the odds of a phenomenon occurring in a given group (the BAV type in which significant coronary stenosis occurred most frequently), relative to the odds of that phenomenon occurring in another group (the other BAV types).

### 2.7. Statistical Methods

Statistical analysis was performed using Statistica 13.1 software (Statsoft, Palo Alto, CA, USA).

Quantitative variables are presented as the median (Me) and interquartile range (IQR), while qualitative variables are presented as numbers and corresponding percentages. Qualitative data were compared using the χ2 test, while Student’s *t*-test or the Mann–Whitney test were used for the comparative analysis of quantitative data. The choice of statistical tests in the comparative analyses of quantitative values depended on their distribution, which was assessed using the Shapiro–Wilk test for normality. For variables not meeting the assumptions of normality or non-parametric variables, the Mann–Whitney test was used. The Kruskal–Wallis test was used to compare three groups of continuous quantitative variables or ordinal variables, while for qualitative variables, the χ2 test for multivariate contingency tables was used. The analysis of relationships between variables was tested using Pearson’s test when the variables were quantitative and met the assumptions of a normal distribution. Spearman’s test was used for ordinal variables and when the distribution of the studied characteristics differed from normal.

A multivariate model was developed using logistic regression analysis. Demographic data and CT-derived parameters were considered during the analysis. Nominal data were typically represented as 0 and 1. A backward conditional stepwise approach was employed to select the parameters included in the final model. During this process, the least significant predictors of significant coronary stenosis were removed first. The parameters that remained in the final model were independently linked to the risk of significant coronary stenosis. The values predicted by the model range from 0 to 1 and represent the probability of significant coronary stenosis. The Hosmer–Lemeshow goodness-of-fit test was used to assess the overall fit of the final model. To evaluate the accuracy of the proposed model, a ROC curve was constructed.

## 3. Results

### 3.1. Characteristics of the Study Group

**Figure 6:**
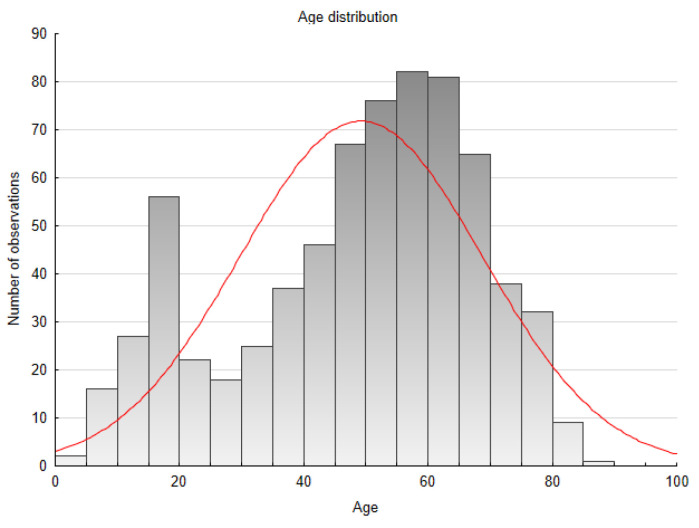
Age distribution of the study population. Normal distribution with a peak at 50 years of age.

**Figure 7:**
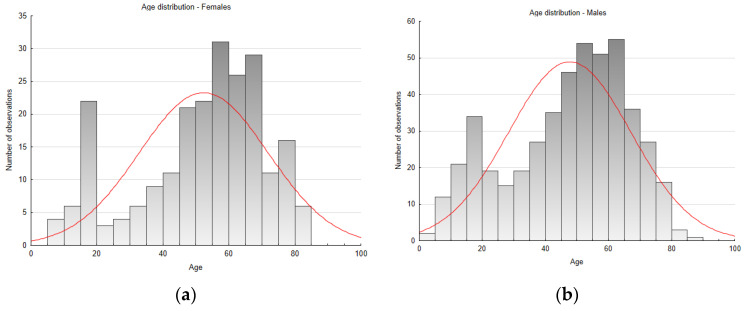
(**a**) Age distribution of females. Left-skewed distribution with a peak at 55 years of age. (**b**) Age distribution of males. Normal distribution with a peak at 50 years of age—similar to the distribution of the whole group.

The study group consisted of 700 patients (473 males—67.6%, 227 females—32.4%). The age range was from 1 to 88 years, with a median of 53 and an IQR of 37–63; in the male group, the age range was 1–88, Me 51, IQR 35–62, while in the female group, the age range was 7–85, Me 57, IQR 42.5–66. The age distributions for the whole study group and by gender are shown below (Figure 6 and Figure 7a,b). Significant age differences were found between males and females (*p* = 0.001).

The prevalence of a bicuspid aortic valve in the group of patients who underwent ECG-gated cardiac CT (*n* = 15,670) was 4.5%.

### 3.2. Structure of the Study Group According to the Sievers–Schmidtke Classification

When sorting the study group based on the Sievers–Schmidtke classification, there were 42 subjects with a type 0 bicuspid aortic valve—6.0% (24 subjects with subtype 0 A-P—3.4% and 18 subjects with subtype 0 lateral—2.6%), 601 subjects with a type 1 bicuspid aortic valve—85.9% (501 subjects with subtype 1 R-L—71.6%, 87 with subtype 1 R-N—12.4%, and 13 with subtype 1 L-N—1.9%), and 57 subjects with a type 2 bicuspid aortic valve—8.1%. The detailed characteristics of the study group, taking the Sievers–Schmidtke division into account, are presented below (Table 1). Statistically significant differences were detected for age between bicuspid aortic valve types (*p* < 0.001). For the other parameters characterizing the study group, no significant differences were observed between BAV types (*p* > 0.05).

### 3.3. Indications for ECG-Gated Cardiac CT

Patients diagnosed or confirmed to have BAV on ECG-gated CT were referred for this examination for a variety of reasons. The percentage of incidental diagnoses of a bicuspid aortic valve was 71.3% (499 patients). The most common reason for referral was suspected coronary artery disease—219 people (31.1%). A dilated ascending aorta in echocardiography with accompanying aortic valve disease or bicuspid aortic valve was the reason for ECG-CT in 105 people (15.0%). In 64 subjects, the reason for referral was the suspicion or postoperative assessment of congenital cardiovascular defects, excluding a BAV (9.1%). Verification of the bicuspid aortic valve presence was the reason for 7.7% (54 subjects) of all referrals, while accurate parametric assessment of acquired aortic valve disease (aortic regurgitation, aortic stenosis) without an initial diagnosis of a BAV was the reason for 7.6% of all referrals (53 subjects). Among the more frequent indications for ECG-gated CT were a dilated ascending aorta in echocardiography without an initial diagnosis of a BAV or aortic valve disease (42 patients—6.0%) and parametric assessment of aortic stenosis and regurgitation coexisting with a BAV (39 patients—5.6%). Less frequent (1.1–2.9%) reasons for referral for ECG-gated CT in this group of patients included assessment of the heart and the aorta before TAVI, as well as diagnosing anomalies before planned ablation, suspicion of aortic dissection, diagnosing arrhythmias, cardiomyopathies, and complications related to cardiac pacemakers, and diagnosing hypertension causes. Reasons for referral for cardiac ECG-CT representing less than 1% of all referrals were grouped together—33 patients (4.7%)—and included diagnosing causes of pulmonary hypertension, suspicion of valvular vegetations, diagnosing reasons for heart failure, and causes of dyspnea. Furthermore, suspected coronary artery disease was found to be a more frequent reason for referral in females than in males (40.5% vs. 26.8%, *p* < 0.001), the same as an assessment of the heart and the aorta before TAVI (5.3% vs. 1.7%, *p* = 0.013). On the other hand, a dilated ascending aorta co-occurring with aortic valve disease or a bicuspid aortic valve was a more frequent reason for referral in males than in females (18.0% vs. 8.8%, *p* = 0.001), the same as accurate parametric assessment of aortic stenosis or aortic insufficiency coexisting with a bicuspid aortic valve (7.0% vs. 2.6%, *p* = 0.021). The frequency of other reasons for referral for cardiac ECG-gated CT was comparable in both groups (*p* > 0.05) (Table 2). 

Taking into account the age of the patients, three age groups were distinguished: group I up to 40 years of age, group II between 41 and 60 years of age, and group III above 60 years of age. It was found that the most common reason for referral in the youngest group (group I) was the diagnosis of congenital cardiovascular disease—49 patients (24.1%), while suspicion of coronary artery disease was the most common reason for referral in the second and third age groups (41–60 years and >60 years), with 113 (41.7%) and 99 (43.8%) subjects, respectively. The most common reason for referral did not differ between males and females in particular age groups (Table 3).

### 3.4. BAV and Accompanying Cardiovascular Defects in Cardiac CT

**Figure 8:**
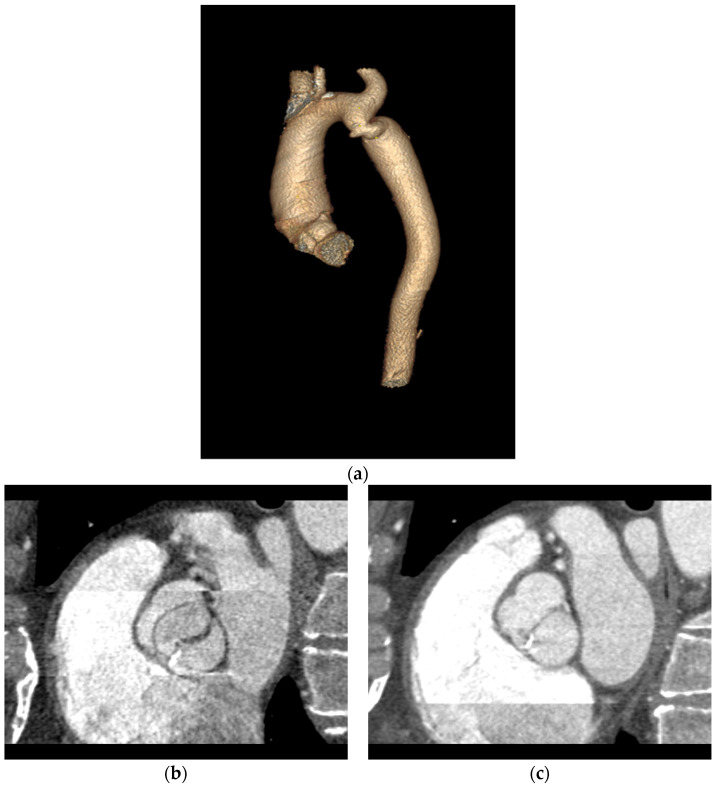
A case of a patient with coarctation of the aorta coexisting with a bicuspid aortic valve (type 0). (**a**) Multislice three-dimensional reconstruction CT scan illustrating coarctation of the aorta; (**b**) type 0 BAV—open; (**c**) type 0 BAV—closed.

The cardiovascular defects most frequently co-occurring with the bicuspid aortic valve included a ventricular septal defect (VSD) (4.3% of the subjects), coarctation of the aorta (3.4% of the subjects) (Figure 8), and an atrial septal defect (ASD) (2.6% of the subjects). In addition, high take-off and a paracommissural orifice of coronary arteries were the two most common coronary anomalies associated with a bicuspid aortic valve (6.4% and 4.3% of the subjects, respectively). Anatomical abnormalities of less clinical significance were more common: patent foramen ovale (PFO) (15.0% of the subjects), and atrial septal aneurysm (7.4% of the subjects). In the group of n = 700 patients with a bicuspid aortic valve, cardiac CT scans revealed 115 clinically significant cardiovascular defects (group I), 111 coronary anomalies (group II), and 284 other cardiovascular abnormalities of minor clinical significance (group III). Details of all cardiovascular abnormalities in patients with a bicuspid aortic valve are shown in Table 4.

**Figure 9:**
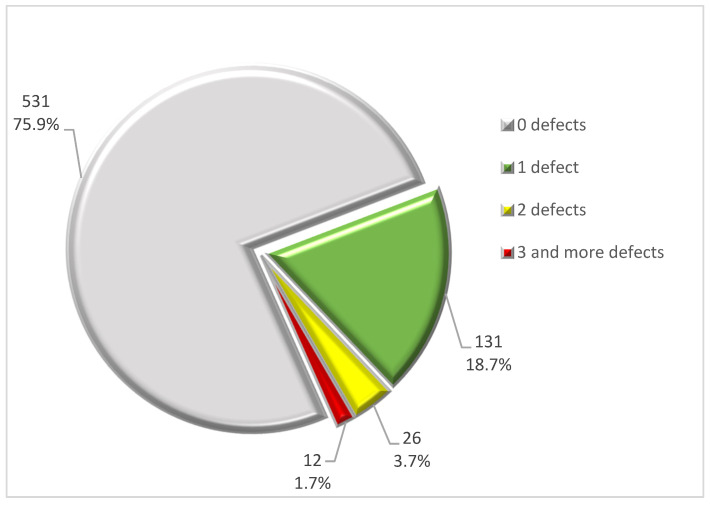
Division of the patient group (n = 700) according to the number of clinically significant cardiovascular abnormalities (group I and II) per subject coexisting with a bicuspid aortic valve on ECG-gated cardiac CT.

Taking into account the number of cardiovascular defects and coronary anomalies (groups I and II, Table 4) accompanying a bicuspid aortic valve, the largest proportion of the group (531 subjects—75.9%) comprised patients with no other cardiovascular defects, apart from the bicuspid aortic valve. In 24.1% of the subjects (169 subjects), a bicuspid aortic valve on cardiac CT was accompanied by at least one clinically significant cardiovascular abnormality. Exactly one clinically significant cardiovascular abnormality was present in 131 subjects with a bicuspid aortic valve (18.7%), two in 26 subjects (3.7%), and three or more in 12 subjects (1.7%) (Figure 9). The ratio of the number of clinically significant cardiovascular abnormalities (groups I and II) per subject was 0.32 for the entire study group (n = 700). Considering only the group of patients with at least one clinically significant cardiovascular abnormality, the value of this ratio was 1.34.

In the female group, the most common cardiovascular defects were a paracommissural orifice of a coronary artery (7.5%), coarctation of the aorta, and high-take-off coronary arteries (4.9% each). In contrast, among males, a high-take-off coronary artery dominated with 7.2%, VSD with 5.3%, and absence of the LMCA with 3.2%. Further analysis showed a comparable number of defects/person between males and females (0.37 vs. 0.30), as well as between separate groups of males and females with clinically significant cardiovascular abnormalities (1.43 vs. 1.29). In accordance with the study methodology, the type of coronary arterial dominance was additionally assessed during the analysis of cardiovascular abnormalities. The percentages of left-dominant coronary circulation in the male and female groups were comparable. Nevertheless, it was observed that in the group of subjects with at least one clinically significant cardiovascular abnormality, left-dominant coronary circulation was more common in females compared to males, but no statistical significance was observed (25.9% vs. 17.1%, *p* = 0.225) (Table 5).

Among clinically significant cardiovascular abnormalities occurring in patients with a bicuspid aortic valve with a prevalence ≥ 1.0%, significant differences were observed between the male and female groups for a paracommissural orifice of a coronary artery and partial anomalous pulmonary venous return (PAPVR)—both defects were more common in females (7.5% vs. 3.0%, *p* = 0.010 and 3.1% vs. 0%, *p* < 0.001). A ventricular septal defect showed a higher prevalence in males (5.6% vs. 2.2%), but this difference was only found to be quasi-significant (*p* = 0.059). As regards the other defects, there were no significant differences between males and females (*p* > 0.05) (Table 5).

Taking into account the division of BAVs into types and subtypes, according to the Sievers–Schmidtke classification, the most common clinically significant defects coexisting with type 0 of a bicuspid valve were a high-take-off coronary artery (11.9%) and coarctation of the aorta (9.5% of the patients); with type 1—high-take-off coronary artery (5.5% of the patients) and paracommissural orifice of a coronary artery (4.8%), and with type 2—high-take-off coronary artery (13.5% of the patients), VSD, and PDA (each defect in 3.8% of the subjects). There were no significant differences in the number of patients with clinically significant cardiovascular abnormalities between BAV subtypes (35.7% vs. 23.8% vs. 19.3%, *p* = 0.146). Nevertheless, the number of defects/person reached higher values in type 0 compared to types 1 and 2 of BAV (0.52 vs. 0.31 and 0.30). In addition, there were no statistically significant differences in the incidence of left-dominant coronary circulation between bicuspid aortic valve types and between separate subgroups of bicuspid valve types consisting of patients with clinically significant cardiovascular abnormalities (*p* = 0.220 and *p* = 0.083, respectively). Detailed data are presented in Table 6.

A similar analysis was carried out with the study group divided by age with simultaneous consideration of gender. Significant differences were observed in the number of patients with clinically significant cardiovascular abnormalities between the group of ≤40-year-olds and the group of >60-year-olds. Moreover, there was a significantly higher number of defects/person in the group of ≤40-year-old patients with clinically significant cardiovascular abnormalities when compared with the other two age groups (1.63 vs. 1.16 and 1.63 vs. 1.19, *p <* 0.001). However, there were no significant differences in the incidence of left-dominant coronary circulation between the groups. Clinically significant cardiovascular abnormalities occurring most frequently in the group of ≤40-year-olds were coarctation of the aorta in females—9 subjects (16.6%), and a high-take-off coronary artery in males—17 subjects (11.4%). In the group of 41–60-year-olds, the most common findings were paracommissural orifice of a coronary artery in females—5 individuals (5.9%), and the absence of the left main coronary artery in males—12 individuals (6.5%). In the third group aged >60 years, the highest prevalence was noticed of a paracommissural orifice of a coronary artery in females—five women (5.7%), and high take-off of a coronary artery in males—seven men (5.1%). Data on the prevalence of other clinically significant cardiovascular abnormalities by age group are presented in Table 7.

### 3.5. Coronary Artery Stenosis in Patients with BAV

**Figure 10:**
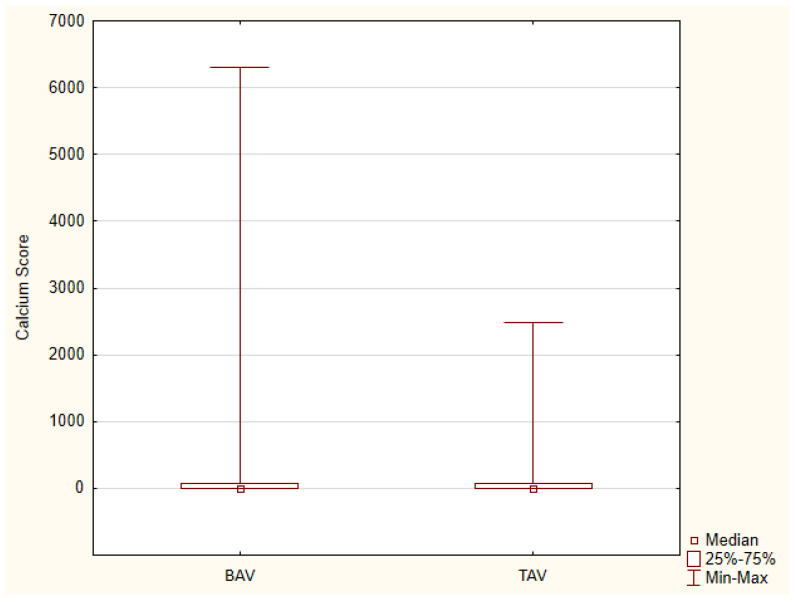
Coronary artery calcium score in patients with bicuspid aortic valve and tricuspid aortic valve—box plot.

We analyzed the calcium score and coronary artery stenosis severity between a group of patients with a bicuspid aortic valve (n = 700, %Males = 67.6%, Age Me = 53) and a control group of patients with a tricuspid aortic valve (n = 100, %Males = 66.0%, Age Me = 52). There was no difference in coronary artery calcium score (CACS) between BAV and TAV (mean 152.2 ± 474.7 vs. 77.4 ± 259.7 U; *p* = 0.150). However, significant coronary artery stenosis (>50%) occurred more frequently in patients with BAV (20.4% vs. 12.0%, *p* = 0.046) (Figure 10, Table 8).

**Figure 11:**
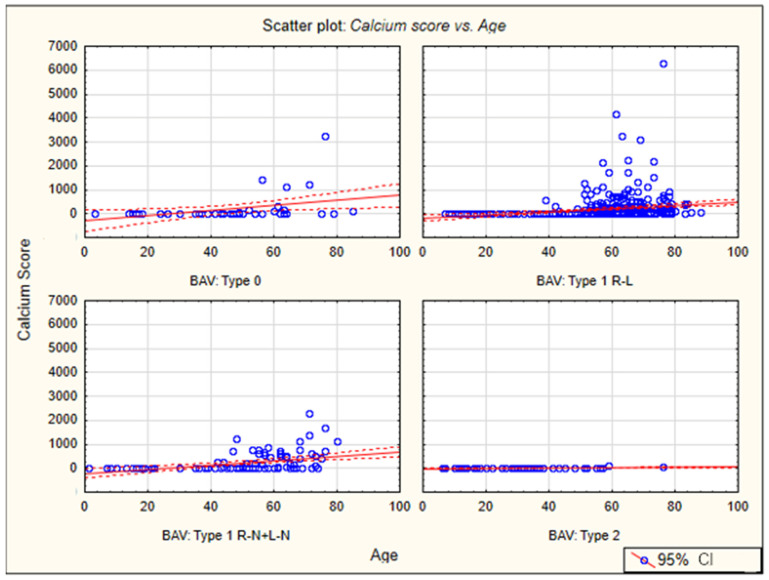
Association of CAC with age for different types of bicuspid aortic valve.

The coronary artery calcium (CAC) scores for all assessed types of BAVs correlated significantly with age (*p* < 0.001). The collected values of these correlation coefficients are shown in Table 9. The lowest correlation coefficients were noted for type 2 of the bicuspid aortic valve (the lowest mean age of all types of BAV), and the highest for type 0 and type 1 R-L. The summary graph shows the relationship between the calcium score and age for the different types of bicuspid aortic valves, with type 1 divided into subtypes 1 R-L and 1 R-N + 1 L-N (Figure 11).

When analyzing the distribution of CAC scores by bicuspid aortic valve types, it was noted that significantly lower values of this index were recorded for type 2 of the BAV compared to the other types of BAVs (*p* < 0.001), with the lowest mean age in this group (29.8 years). The distribution of coronary calcium score values in the other valve types was comparable, with the highest values at the 75th and 90th percentile in the 1 R-N + 1 L-N group, as shown in Table 10. The highest calcium score values (95th percentile) were recorded for type 0 (1230.6 U) and for type 1N-R-1N-L (1115.7 U). In addition, the highest increase in CAC score among all BAV types was reported between the 90th and 95th percentile for type 0 (an increase of 936.2 U). For all types of bicuspid aortic valves, the 50th percentile value was 0, so at least half of the subjects in each type of bicuspid aortic valve had no coronary artery calcification. In type 2 of the BAV, a CAC score of zero characterized the 75th percentile.

To complement this, the analysis was supplemented with the distribution of CAC scores by age and gender. In the group of ≤40-year-olds, the percentage of subjects without coronary artery calcifications was 97% (95th percentile value equal 0) and was comparable between males and females. In the group of 41–60-year-olds, 55.7% of the subjects had no coronary artery calcifications, while in the group of >60-year-olds, this percentage decreased to 27.9% of the subjects. In both the 41–60-year-old and >60-year-old groups, the mean Agatston score was significantly higher in males than in females (*p* < 0.001 for the 41–60-year-old group and *p* = 0.013 for the >60-year-old group), as illustrated in Table 11. In the group of 41–60-year-olds, the median CAC score (50th percentile) was 0, while in the group of >60-year-olds, the value of 0 was only observed up to the 25th percentile (irrespective of gender for both age groups). It is noteworthy that the 75th percentile for calcium score in the II and III age groups was slightly higher in females than in males. In contrast, the 90th and 95th percentile values of calcium score were higher in males in both age groups (G.II and G.III).

In the Kruskal–Wallis test, significant differences in the coronary artery stenosis severity were observed between groups (*p* < 0.001), as shown in Table 12. A post hoc analysis showed that significant differences in the coronary artery stenosis severity were present between type 2 and each of the other bicuspid aortic valve types. In addition, significant differences were noted between type 1 R-N + 1 L-N and type 1 R-L (33.0% vs. 18.4%, *p* = 0.001), as well as between type 1 R-N + 1 L-N and type 0 of the BAV (33.0% vs. 16.7%, *p* = 0.048).

**Figure 12:**
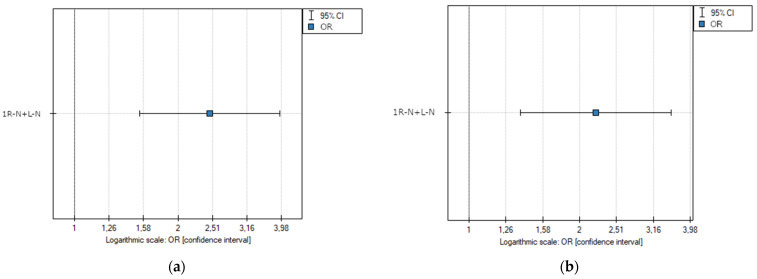
(**a**) Odds ratio plot with a confidence interval of significant coronary stenosis resulting from univariate logistic regression (n = 700). (**b**) Odds ratio plot with a confidence interval of significant coronary stenosis resulting from univariate logistic regression (n = 643)—type 2 BAV excluded. The presence of a raphe between the coronary and non-coronary cusps was considered a risk factor for significant coronary artery stenosis.

Subsequently, a univariate logistic regression analysis was performed on a group of n = 700 individuals (100 subjects with subtype 1 R-N + 1 L-N—a single raphe between the coronary and non-coronary cusps and 600 subjects with the other types of BAVs), which showed that statistically significant predictive parameters indicating the presence of significant coronary artery stenosis (≥50%) included the presence of a single suture between the coronary and non-coronary cusps (1 R-N + 1 L-N) (OR 2.46 [1.54–3.94]; *p* < 0.001) (Figure 12a).

Due to significant age differences between the subjects with type 2 of the bicuspid aortic valve and each of the other types of bicuspid aortic valve, a univariate logistic regression analysis was performed with the exclusion of patients with type 2 of the BAV (n = 57). This yielded a group of n = 643 individuals (100 patients with subtype 1 R-N + 1 L-N—a single raphe between coronary and non-coronary cusps, and 543 patients with the other types of BAV). The analysis also showed that statistically significant predictive parameters influencing the occurrence of significant coronary artery stenosis (≥50%) included the presence of a single raphe between the coronary and non-coronary cusps (1 R-N + 1 L-N) (OR 2.20 [1.38–3.53]; *p* < 0.001) (Figure 12b).

To confirm the predictive power for significant coronary artery stenosis of a BAV type and its independent association, a multivariate analysis was conducted. Two groups were separated: the first consisted of 143 patients with significant coronary stenosis, while the second group included 557 patients without significant stenosis in the coronary arteries.

Initially, a univariate analysis was performed on a group of 700 subjects, indicating that the predictive parameters significantly influencing the occurrence of significant coronary artery stenosis include age, gender, calcium score, and type of BAV, as presented in Table 13.

**Figure 13:**
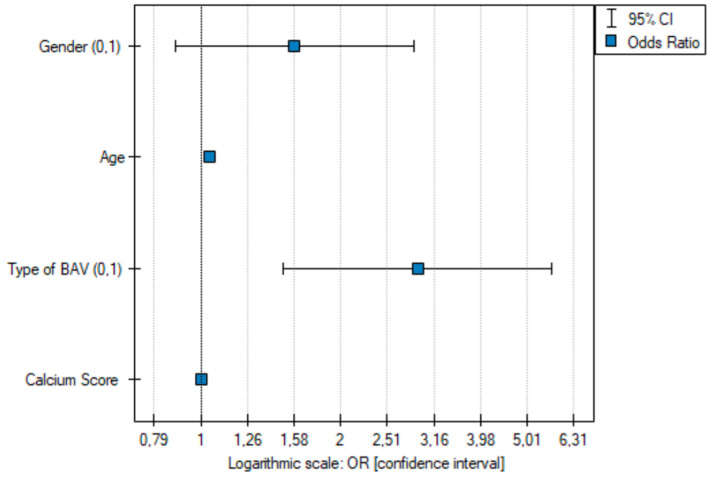
Odds ratio plot with a confidence interval of significant coronary stenosis resulting from multivariate logistic regression (n = 700). Data encoding gender (0—females, 1—males) and type of BAV (0—other types of BAV, 1—type 1 R-N + 1 L-N).

**Figure 14:**
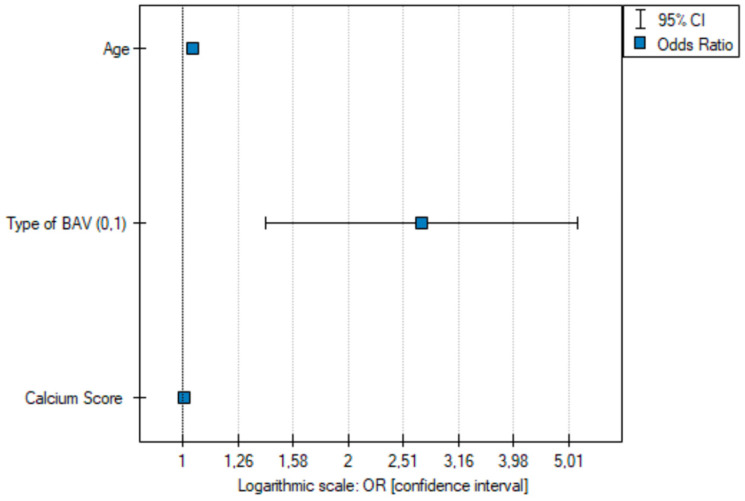
Odds ratio plot with a confidence interval of significant coronary stenosis resulting from multivariate logistic regression (n = 700)—after excluding an insignificant variable (gender). Data encoding gender (0—females, 1—males) and type of BAV (0—other types of BAV, 1—type 1 R-N + 1 L-N).

After univariate analysis, multiparameter analysis was performed using the backward conditional stepwise method. The BAV type, age, and calcium score were found to be statistically significant predictors of significant coronary artery stenosis (Figure 13 and Figure 14).

Details of the logistic regression analysis are presented in Table 14. The obtained model for identifying significant stenosis in coronary arteries is as follows:

z = −4.926 + 0.042 × AGE + 0.997 × BAV TYPE + 0.006 × CALCIUM_SCORE

**Figure 15:**
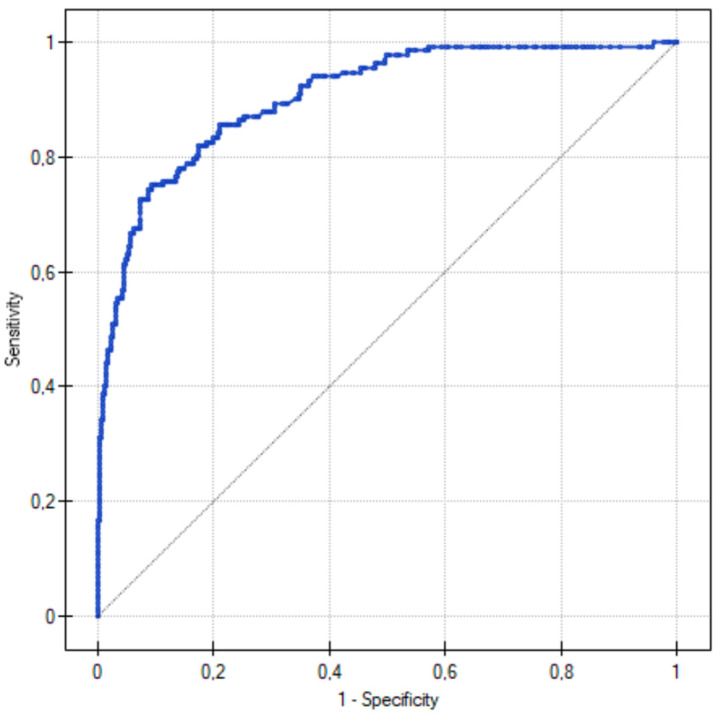
ROC curve for the obtained model. Area under the curve = 0.905, 95% CI: 0.877–0.934.

The proposed cut-off point for the ROC curve was 0.146, which was lower than the standard level of 0.5 used in the regression. The obtained predictive model had the highest sensitivity and specificity of 0.82 and 0.82, respectively. The ROC curve of the probability of significant coronary artery stenosis calculated by the model is shown in Figure 15. The Hosmer–Lemeshow test showed a value of 12.907 (*p* = 0.115), which indicated a good fit of the model.

## 4. Discussion

Multislice CT is commonly used in the assessment of the coronary arteries, while the heart valves are routinely examined with echocardiography. In recent years, the importance of CT scanning as well as the number of indications for this examination have been increasing. According to the 2012 European Society of Cardiology guidelines for the management of valvular heart disease, MSCT can contribute to the assessment of the severity of aortic valve disease either indirectly by quantifying valve calcification, or directly by measuring valve planimetry [57].

The analysis of indications for referral for ECG-CT in patients with a BAV is particularly substantial from the radiologists’ perspective. Taking into account the relatively high frequency of BAV in the group of patients who underwent ECG-CT for various reasons (499/15,670–3.2%), we can expect an incidentally diagnosed bicuspid aortic valve in approximately every 30th patient examined with cardiac CT. Due to the frequent occurrence of an incidental BAV, it seems reasonable to use appropriate reconstruction techniques that show the full dynamics of valve movement. However, this is not a routine and obligatory procedure, for example, in patients diagnosed with coronary artery disease, who constitute a significant percentage of those referred for ECG-CT. It should be emphasized that the phases of aortic valve opening should always be assessed, regardless of the indications for ECG-CT. In the diastolic phase, only a type 0 BAV can be diagnosed. ECG gating becomes particularly useful in the diagnosis of valves with a residual raphe between the cusps, which typically require data acquisition during both systole and diastole of the heart. It is also difficult to determine the presence or absence of a raphe in TTE. However, if a bicuspid aortic valve is suspected on referral, radiologists should evaluate this area carefully, including the systolic and diastolic phases. Our study shows that it seems reasonable to evaluate the aortic valve in multiple reconstructions taking into account its full opening and closing, not only in patients with a suspected BAV, but in all patients referred for cardiac ECG-CT.

In our study, we used the Sievers–Schmidtke classification, which was adopted by our department for cardiac computed tomography and has been routinely used for many years. Nevertheless, it should be emphasized that since 2021, there has been an International Consensus statement on nomenclature and classification of the congenital bicuspid aortic valve, published by Michelena et al. [17]. This highlights some limitations of the Sievers classification such as the lack of prerepair symmetry assessment, inability to define all BAV phenotypes, and lack of recognition of aortopathy phenotypes. Furthermore, it recognizes partial fusion between cusps of a BAV and does not include unicuspid aortic valves (type 2 of BAV). Michelena et al. distinguished three BAV types: the fused BAV, the two-sinus BAV, and the partial-fusion BAV, each with specific phenotypes. The fused type partially corresponds to type 1 according to the Sievers–Schmidtke classification, while the two-sinus BAV partially corresponds to type 0 of the BAV. Among fused BAVs, three phenotypes are described: right–left cusp fusion, right non-(non-coronary) cusp fusion, and left non-(non-coronary) fusion, which partially correspond to subtypes 1 R-L, 1 R-N, and 1 L-N according to the Sievers–Schmidtke classification [16]. Additionally, a study by Michelena et al. defines that fused types may have raphe or not, while two-sinus types do not have a raphe. In contrast to the study by Michelena et al., type 0 in the Sievers classification does not differentiate between a fused BAV with no raphe and a two-sinus BAV [16,17]. The classification proposed by Sievers–Schmidtke does not include symmetry assessment, while in a study by Michelena et al., symmetry of a fused BAV was taken into account. Assessment of BAV symmetry for the fused BAV type is determined by the angle formed between the commissures of the non-fused cusp. A symmetrical fused BAV is defined for an angle of 160–180 degrees. As the angle between the commissures of the non-fused cusp decreases to less than 160°, the BAV becomes asymmetrical [17]. There are also two major phenotypes of the bicuspid aortic valve aortopathy. The first phenotype is the ascending phenotype, characterized by dilation mainly located in the tubular ascending tract beyond the sino-tubular junction. The root phenotype features predominant dilation of the root [17]. The association of the bicuspid aortic valve and aortopathy has been comprehensively described, both based on echocardiography and computed tomography, and therefore we have not delved into this issue and we have not used the classification by Michelena et al. Most of the studies published so far assessing the bicuspid aortic valve on CT used the Sievers–Schimdtke classification; therefore, it seems reasonable to use the same classification in terms of comparability of results. It should be emphasized that we did not include patients with a partial-fusion BAV (less than 50% cusp fusion) in our study. The prevalence of this recently recognized partial-fusion BAV is unknown [58] and impossible to compare with other studies. “Mini-raphes” that did not meet the criterion of at least 50% cusp fusion and did not present the opening shape typical of a bicuspid aortic valve were also not analyzed in this study and were recognized only as a variant similar to a bicuspid aortic valve. The same approach is used by echocardiographists at our hospital. Furthermore, it has been common practice in our country to classify the unicuspid aortic valve as a bicuspid aortic valve (type 2 of the BAV). Nevertheless, we are in the process of adapting the classification by Michelena et al. to meet the needs of new patients who are being qualified by cardiac surgeons for surgical procedures.

To date, there have been very few studies evaluating the morphology of the bicuspid aortic valve on CT. Szymczyk and co-authors identified a group of 19 patients with bicuspid aortic valves from a population of 2053 patients who underwent multislice CT. In addition, they adapted the Sievers–Schmitke classification assessing the bicuspid aortic valve morphology on CT. A bicuspid aortic valve was noted in 0.9% of the study participants (19 subjects), among whom five had type 0 (0.2%) and 14 had type 1 (0.7%). Type 2 of the BAV was not found in any of the subjects. Nevertheless, this study did not include an analysis of defects coexisting with the bicuspid aortic valve. The focus was mainly on assessing the function of the bicuspid aortic valve, as well as measuring the diameter of the ascending aorta in patients with different types of BAVs compared to patients with tricuspid and quadricuspid aortic valves [59]. In a study by Yoon et al., a group of 1,070 patients with a bicuspid aortic valve who underwent a TAVR procedure were evaluated in a CT scan. A modified Sievers classification was used to assess the BAV morphology, specifying type 0—without the presence of a raphe, and type 1—with the presence of a raphe between the BAV cusps. Type 0 was present in 10.3% of the subjects, while type 1 was present in 89.7%. The study analyzed whether the valve type, the presence of calcification at the raphe, and calcification within the valve cusps affect mortality at 2 years after the procedure. A multivariate analysis showed that the type of BAV (presence or absence of raphe) did not affect the prognosis of patients after TAVR, while a significant effect was noted for the presence of calcification at the raphe and calcification on the bicuspid aortic valve cusps [60]. Another study by Zhang et al. assessed whether the presence or absence of a raphe in patients with BAV influences the degree of valve dysfunction and type of aortopathy. They studied 312 patients from the Korean population with bicuspid aortic valves who underwent both cardiac CT and echocardiography to assess the BAV morphology. It was found that the presence of a raphe was significantly associated with a higher incidence of aortic regurgitation, a lower incidence of aortic stenosis, and less dilatation of the aortic root and the middle ascending aorta [61]. The use of cardiac computed tomography to diagnose a bicuspid aortic valve was also described in Uhlig S’s doctoral dissertation. It evaluated the BAV morphology, valvular complications of a bicuspid aortic valve, and congenital cardiovascular defects coexisting with BAV, but without considering their incidence in each type of BAV. It also did not address coronary artery stenosis in patients with particular types of BAVs [62].

A bicuspid aortic valve is often accompanied with additional defects of the heart, blood vessels, and other organs, and their coexistence is attributed to genetic mutations in the genes responsible for embryogenesis of both the aortic valve and other heart structures [8]. The most common defects associated with a BAV are coarctation of the aorta and septal defects. Nevertheless, little is known about the prevalence of other cardiovascular abnormalities in patients with bicuspid aortic valves. In our study, we noted that clinically significant defects most commonly co-occurring with a bicuspid aortic valve were VSD (4.3% of the patients) and coarctation of the aorta (3.6% of patients). These were slightly more common in patients with type 0 of the bicuspid aortic valve, but the significance of this relationship was not assessed due to small numbers of these defects in the particular subgroups of bicuspid aortic valve cases. In the general population, VSD is the most common heart defect in neonates, where it occurs with a prevalence of 2–5%. In adults, it is much less common [2]. In our study, VSD was the most common finding on CT scans in patients ≤ 40 years of age with a prevalence of 6.4%. In addition, we found a higher prevalence of VSD in the male population with a bicuspid aortic valve compared to females (5.3% vs. 2.2%, *p* = 0.059—quasi-significant result). An assessment of the prevalence of VSD in patients with BAVs was also described in the study by Uhlig—it was found in 4.3% of the patients [62]. On the other hand, coarctation of the aorta represents the fourth most common cardiovascular defect occurring in 7% of patients with congenital heart defects [63]. According to some studies, the detection rate of BAVs in patients with coarctation of the aorta is over 50% and is most often associated with the presence of subtype 1 R-L of the BAV [9,64]. Our results indicated that coarctation of the aorta was more frequently identified in patients with type 0 BAVs on cardiac CT—in 9.5%. In addition, there were no significant differences in the prevalence of coarctation of the aorta between females (4.9%) and males (3.0%). A higher prevalence (7.5%) of coarctation in patients with a BAV, compared to our study, was obtained by Uhlig S. in his doctoral dissertation, but his study was conducted on a smaller patient group [62].

Much less is known about the prevalence of other cardiovascular abnormalities in patients with BAVs. The prevalence of ASD in the general population is estimated to be 56/100,000 live births [2]. In some cases, this defect is asymptomatic for many years, resulting in it being detected in adults, accounting for 7–12% of all heart defects in this patient group [11]. In our study, it occurred in 3.0% of the patients with a BAV (a similar result was obtained by Uhlig S.—2.6%), most commonly in type 0 of the bicuspid aortic valve, with a prevalence of 4.8%. Patent foramen ovale is another form of atrial septal defect of less clinical significance. It is seen in 15–35% of the general population and is associated with failure of the fetal foramen ovale to close [65]. In our group of patients with a bicuspid aortic valve, PFO was present in 15.0% of the patients, which is comparable to the general population. Our study was the first to assess the prevalence of clinically significant cardiovascular defects on CT in patients with a BAV, taking into account the type of bicuspid aortic valve, as well as the age group. Patients with type 0 of the BAV had a higher number of defects/person compared to type 1 and type 2 (0.52 vs. 0.31 and 0.30), indicating that more than one clinically significant cardiovascular defect or congenital syndrome occurred more frequently in type 0 than in other types of BAVs. A similar relationship was noted for the ≤40-year-old group compared to the other two age groups, indicating that a large proportion of clinically significant cardiovascular defects coexisting with the BAV on CCTA were diagnosed in younger individuals, particularly those with a type 0 bicuspid aortic valve.

The prevalence of coronary anomalies in the general population is estimated to be 0.3–2% [66,67,68,69]. To date, there have been very few papers characterizing coronary anomalies in patients with bicuspid aortic valves. In our group of patients with BAVs, the presence of at least one coronary anomaly on CT scans was found in 98 patients (14.0%). Fedak et al. mention that the common occurrence of BAVs and coronary artery anomalies may have a genetic basis [70]. In a study by Michalowska and co-authors based on CCTA results, the prevalence of coronary artery anomalies in patients with BAVs was 2.6% (4/193), slightly higher than that in the tricuspid aortic valve group (1.7%), *p =* 0.05. In addition, Michalowska found that the prevalence of LMCA absence was significantly higher in patients with BAVs (7.3%–14/193) compared to patients with tricuspid aortic valves (TAVs) (1.7%–4/235), *p* = 0.004 [71]. Koeenradt et al. indicated that the frequency of LMCA absence was significantly higher for type 0 (A-P) of the bicuspid aortic valve compared to type 1 (*p =* 0.047) [72]. A study by Naito et al. assessed the frequency of coronary anomalies in patients with bicuspid and tricuspid aortic valves using data from echocardiography, cardiac computed tomography, and magnetic resonance imaging. Coronary anomalies were divided into three main groups: anomalous ostium of the right coronary artery (RCA), anomalous origin of the LMCA, and separate ostia of the left anterior descending artery/cirumflex artery (LAD/LCx). The aforementioned groups were subdivided into smaller subgroups of coronary anomalies, but their incidence was recorded for the entire study group, without specifying patients with a BAV or TAV. The calculations specifying patients with a BAV and TAV were performed only for the main groups of coronary anomalies. Separate ostia of LAD/LCx were found in 2% of the subjects with a BAV, an anomalous ostium of the RCA in 4% of the subjects, and an anomalous origin of the LMCA in 0.9% of BAV subjects. Furthermore, an anomalous ostium of the RCA and separate ostia of LAD/LCx were found to be significantly more frequent in patients with BAV compared to patients with TAV (*p =* 0.005 and *p =* 0.031, respectively) [73]. According to a study by DeFaria et al., a bicuspid aortic valve is characterized by an increased incidence of separate left coronary ostia, high-take-off coronary arteries, and left-dominant system [74]. In addition, an anomalous coronary origin from the opposite coronary sinus is three times more common in patients with a BAV, compared to those with a TAV [75]. The most common coronary anomalies in our study included an anomalous origin of coronary arteries. Among them, high take-off (6.4% of the subjects) and paracommissural orifice coronary arteries were distinguished (4.4% of subjects). However, category A2a (high take-off) and category A3b (origin from the ascending aorta), according to the Angelini classification, were combined into a common category of high-take-off coronary arteries in our study, which influenced its higher percentage in the group of patients with a BAV. In males, the most common coronary anomaly identified in patients with a BAV on CT scans was high-take-off coronary arteries (7.2% of the subjects), while in females, it was a paracommissural orifice of coronary arteries (7.5% of the subjects). Furthermore, a paracommissural orifice was one of the few cardiovascular abnormalities more common in females than in males among the subjects with bicuspid aortic valves (7.5% vs. 3.0%, *p* = 0.010). PAPVR was another one, which was present in only seven women in our study. As reported by Ho et al., PAPVR is only slightly more common in females in the general population, constituting 58% of all PAPVR cases [76]. In our study group, we additionally found that high-take-off coronary arteries were significantly more frequent in patients with type 2 of the BAV compared to type 1 (*p* = 0.04).

It is worth mentioning that 102 patients with a bicuspid aortic valve (14.6%) had a left-dominant coronary circulation. In the study by Michalowska and co-authors, the incidence of left-dominant systems in patients with a BAV was 15.5% (30/193), while it was lower in patients with a tricuspid valve—8.9% (21/235), *p* = 0.08 [73]. In the general population, left-dominant coronary circulation is found in 8–13% of patients [66]. A higher prevalence of left-dominant coronary circulation in patients with a BAV was also reported by DeFaria [74]. The study by Zhou et al. demonstrated that patients with a bicuspid aortic valve and left-dominant coronary circulation had a significantly higher incidence of coarctation of the aorta compared to patients with right-dominant hearts (44% vs. 14%, *p* = 0.04) [77]. Our study showed that patients with type 0 of the BAV (in whom congenital cardiovascular abnormalities represent a significant proportion of all abnormalities) were more likely to have a left-dominant coronary circulation compared to types 1 and 2 (40.0% vs. 17.5% vs. 27.3%), and this relationship turned out to be statistically insignificant (*p* = 0.083). The same finding was made in the study by Koenraadt et al., who found that type 0 of the BAV (A-P) was significantly more often associated with a left-dominant coronary circulation compared to type 1 of the BAV (*p =* 0.047) [72]. In contrast, such an association was not confirmed by Szymczyk et al., who noted no differences between bicuspid aortic valve types in coronary arterial dominance, but the group of patients with a BAV in their study included only 19 patients [59].

Previously published studies have compared the incidence of coronary artery disease in patients with bicuspid and tricuspid aortic valves, but have not analyzed the different types of BAVs, nor with gender and age considerations, which were included for the first time in our study. A meta-analysis by Poggio et al. showed that patients with a bicuspid aortic valve were less likely to develop coronary artery disease compared to those with a tricuspid one. Nevertheless, this may have been related to the lower mean age and lower prevalence of hypertension and diabetes in this group [78]. Feuchtner et al. demonstrated that patients with a bicuspid aortic valve had a lower coronary artery calcification index compared to those with a tricuspid aortic valve (237.4 vs. 1013.3AU; *p* < 0.001), and a lower coronary artery stenosis severity (CAD-RAD™: *p* < 0.001) [79]. In our study, we did not find differences in the coronary artery calcium score (CACS) between BAVs and TAVs (mean 152.2 ± 474.7 vs. 77.4 ± 259.7 AU; *p* = 0.150). However, significant coronary artery stenosis (>50%) occurred more frequently in patients with BAVs (20.4% vs. 12.0%, *p* = 0.046). In the studies published to date, differences between bicuspid aortic valve types and subtypes have only been sought in terms of the prevalence of aortic stenosis and regurgitation. The 1 R-L subtype was shown to be more prone to developing aortic regurgitation, whereas 1 R-N showed a higher likelihood of aortic stenosis [21,80]. A study by Szymczyk et al. found that there were no significant differences in the incidence of coronary artery disease between the different types of BAVs [59]. On the other hand, Koenraadt et al. found that type 0 (A-P) was significantly more frequently associated with the incidence of coronary artery disease compared to type 1 of the BAV (*p =* 0.047) [72]. However, none of the authors mentioned above addressed the differences in calcium scores or significant coronary stenosis between the different types of BAVs.

In our study, we showed that significant differences in CAC scores were present between type 2 of the bicuspid aortic valve and each of the other types (*p* = 0.001). For both type 0 and type 1 of the BAV, the percentage of patients without calcification in the coronary arteries oscillated around 60%, while for type 2, the percentage was around 85%. Nevertheless, the mean age in this group was the lowest at 29.8 years. The mean age in the 41–60 age group of males was 51 years and, according to the MESA calculator, the 50th percentile value for this age group was 0, which was the same as for the mean age in the group of males aged 41–60 years old with a bicuspid aortic valve. A slightly higher value in our group was reported for the 75th percentile compared to that calculated based on the MESA study for the general population (61 vs. 29). In the group of 41–60-year-old females with BAV, the mean age was 52 years. The 50th percentile value for this age was 0, the same as that calculated with the MESA calculator for the general female population. In contrast, the 75th percentile values were much higher (78 vs. 0). A similar analysis was performed in the >60 age group. The mean age of the males in this group was 68 years. The 50th percentile value for this age was 83, which was slightly lower than the value calculated with the MESA calculator—119. For the 75th percentile, a value of 509 was obtained, which was slightly higher than the value calculated for the general population of males of this age—447. A similar analysis was performed for the group of females with bicuspid aortic valves aged >60—the mean age was 70 years. Both the 50th and 75th percentile values for this age were significantly higher than those calculated using the MESA calculator (83 vs. 13 for the 50th percentile and 547 vs. 119 for the 75th percentile, respectively). The analysis highlights the significantly higher CAC scores in females over 40 years old with a bicuspid aortic valve compared to the general population. These differences manifest themselves, particularly from the 75th percentile of the calcium score distribution. For the group of males with a bicuspid aortic valve, no differences in calcium score were observed compared to the general male population. However, it should be taken into account that this analysis was performed based on the mean age for each of the age groups mentioned, which may involve a small statistical error.

As for the prevalence of significant coronary artery stenosis (≥50%), significant differences were also noted between type 2 and each of the other bicuspid aortic valve types, as well as between type 1 R-N + 1 L-N and type 1 R-L (*p* = 0.001), and between type 1 R-N + 1 L-N and type 0 (*p* = 0.048). Due to the small number of subtype 1 L-N individuals, we combined them with subtype 1 R-N for a reliable comparative analysis. The low calcium score and coronary artery stenosis severity in patients with type 2 of the bicuspid aortic valve may be due to their BAV-related symptoms occurring early in life, resulting in imaging diagnostics being initiated at a relatively young age. It is worth mentioning that the mean ages in the other types of bicuspid aortic valves were comparable. In addition, no significant differences in gender structure were found between each type of BAV. A sequential univariate regression analysis indicated that a single raphe between the coronary and non-coronary cusps (1 R-N + 1 L-N) significantly increased the risk of significant coronary stenosis (OR 2.46 [1.54–3.94]; *p* < 0.001) in patients with the bicuspid aortic valve. To increase the reliability of our analysis, a second regression analysis was performed excluding patients with type 2 of the BAV—a group that differed significantly from the other BAV types in terms of age. We confirmed that the presence of a single raphe between the coronary and non-coronary cusps (1 R-N + 1 L-N) significantly increased the risk of significant coronary stenosis (OR 2.21 [1.38–3.54]; *p* < 0.001). A multivariate logistic regression confirmed that a single raphe between the coronary and non-coronary cusps was a predictor of significant coronary stenosis, independently of age and gender.

Our study was limited by its retrospective nature. Prospective studies conducted on large groups of patients may help assess the utility of the predictive factor we describe. In addition, our study did not take into account medications taken by patients permanently for cardiovascular disease, such as acetylsalicylic acid (ASA), statins, and novel oral anticoagulants (NOACs), due to a lack of data. We also did not compare the efficacy of CTA with the efficacy of invasive coronary angiography in the assessment of coronary stenosis. This is because CTA tends to overestimate the severity of stenosis when massive coronary artery calcification is present and in patients with a CAC score > 400 units [81].

## 5. Conclusions

The presence of a raphe between the coronary and non-coronary cusps of a bicuspid aortic valve appears to be an independent predictor of significant (≥50%) coronary stenosis, but there is a need for further studies conducted on a larger patient group, including multivariate regression models, to confirm this relationship.

The low mean age of patients with type 2 of a bicuspid aortic valve and the low coronary artery stenosis severity, as well as low CAC scores in this group, were mainly due to the fact that symptoms associated with a BAV appear early in life in those patients, which influences the initiation of diagnostics aimed at assessing the valve of the aortic orifice at a younger age.

Cardiovascular defects co-occurring with bicuspid aortic valves are characterized by varying prevalences according to age, gender, and valve type, as defined by Sievers–Schmidtke.

In patients ≤ 40 years old, as well as those with type 0 of the bicuspid aortic valve, cardiovascular defects, such as coarctation of the aorta and VSD, were more frequently found on ECG-gated CT. In patients > 40 years old, as well as those with type 1 and 2 of the bicuspid aortic valve, there was a significantly increased percentage of coronary anomalies among the ECG-CT-diagnosed abnormalities co-occurring with the bicuspid aortic valve.

The incidence of clinically significant cardiovascular abnormalities in patients with bicuspid aortic valves was independent of gender, except the paracommissural orifice of coronary arteries and PAPVR.

Regardless of gender, the most common reason for referral for cardiac ECG-gated CT in the group of young people (<40 years old) with a bicuspid aortic valve was the diagnosis of congenital cardiovascular defects, while in the group of over 40-year-olds, the most common reason was a suspicion of coronary artery disease.

## Figures and Tables

**Table 1:** Characteristics of the study group with its division by bicuspid aortic valve types, according to the Sievers–Schmidtke classification. Quantitative parameters are presented as median (Me), minimum (Min), and maximum (Max), while qualitative parameters are presented as absolute value (n) and corresponding percentage (%). * Characteristics of type 1 bicuspid aortic valve are divided into subtypes 1 R-L and 1 R-N + 1 L-N.

Characteristics of the Study Group					
	Type 0 (n = 42)	Type 1 R-L * (n = 501)	Type 1 R-N + 1 L-N * (n = 100)	Type 2 (n = 57)	*p*
Age [years], Me (Min–Max)	48.5 (3–85)	56 (7–88)	54 (1–80)	30.5 (6–76)	<0.001
Sex–Males, n (%)	30 (71.4%)	342 (68.3%)	60 (60.0%)	41 (71.9%)	0.320
BMI, Me (Min–Max)	26.1 (16.0–39.1)	28.1 (11.3–41.9)	26.6 (15.6–38.4)	24.8 (15.4–39.7)	0.070
Ejection fraction [%], Me (Min–Max)	59 (33–87)	62.5 (16–85)	63 (16–83)	63.5 (19–78)	0.236
End-diastolic volume [ml], Me (Min–Max)	164.5 (60–453)	172 (59–508)	159 (12–643)	174 (57–486)	0.258
Dyslipidemia, n (%)	20 (47.6%)	221 (44.1%)	48 (48.0%)	25 (43.9%)	0.882
Diabetes, n (%)	4 (9.5%)	54 (10.8%)	16 (16.0%)	6 (10.5%)	0.481
Atrial fibrillation, n (%)	10 (23.8%)	75 (15.0%)	10 (10.0%)	6 (10.5%)	0.145
Hypertension, n (%)	10 (23.8%)	119 (23.8%)	22 (22.0%)	13 (22.8%)	0.984

**Table 2:** Reasons for referral for cardiac ECG-gated CT in patients with bicuspid aortic valve by gender. * Diagnosing causes of pulmonary hypertension, suspicion of valvular vegetations, diagnosing reasons for heart failure, and causes of dyspnea.

Indications for ECG-Gated Cardiac CT in Patients with BAV	Overall (n = 700), n (%)	Males (n = 473), n (%)
1. Suspected coronary artery disease	219 (31.1%)	127 (26.8%)
2. Suspicion or postoperative assessment of congenital cardiovascular defects excluding BAV	64 (9.1%)	40 (8.5%)
3. Verification of BAV presence	54 (7.7%)	40 (8.5%)
4. Dilated ascending aorta in echocardiography without initial diagnosis of BAV or aortic valve disease	42 (6.0%)	31 (6.6%)
5. Dilated ascending aorta in echocardiography with accompanying aortic valve disease (aortic stenosis, aortic insufficiency) or bicuspid aortic valve	105 (15.0%)	85 (18.0%)
6. Suspicion of aortic dissection	12 (1.7%)	6 (1.3%)
7. Parametric assessment of acquired aortic valve disease (aortic stenosis, aortic insufficiency) without initial diagnosis of BAV	53 (7.6%)	39 (8.2%)
8. Parametric assessment of aortic stenosis or aortic insufficiency coexisting with BAV	39 (5.6%)	33 (7.0%)
9. Diagnosing for cardiomyopathy causes	11 (1.6%)	6 (1.3%)
10. Diagnosing for hypertension causes	8 (1.1%)	2 (0.4%)
11. Diagnosing for arrhythmia—suspicion of arrhythmogenic right ventricular dysplasia	12 (1.7%)	10 (2.1%)
12. Diagnosing for complications related to cardiac pacemakers	10 (1.4%)	5 (1.1%)
13. Diagnosing before planned ablation	18 (2.6%)	15 (3.2%)
14. Assessment of the heart and the aorta before transcatheter aortic valve implantation (TAVI)	20 (2.9%)	8 (1.7%)
15. Others *	33 (4.7%)	26 (5.5%)

**Table 3:** Reasons for referral for ECG-gated CT in patients with a bicuspid aortic valve by age group and gender of subjects.

The Most Common Indications for ECG-Gated Cardiac CT					
Patients with BAV in ECG-Gated Cardiac CT (n = 700)					
≤40 years (n = 203)	n (%)	41–60 years (n = 271)	n (%)	>60 years (n = 226)	n (%)
Suspicion or postoperative assessment of congenital cardiovascular defects excluding BAV—category 2	49 (24.1%)	Suspected coronary artery disease—category 1	113 (41.7%)	Suspected coronary artery disease—category 1	99 (43.8%)
Dilated ascending aorta in echocardiography with accompanying aortic valve disease (aortic stenosis, aortic insufficiency) or bicuspid aortic valve—category 5	32 (15.8%)	Dilated ascending aorta in echocardiography with accompanying aortic valve disease (aortic stenosis, aortic insufficiency) or bicuspid aortic valve—category 5	48 (17.7%)	Dilated ascending aorta in echocardiography with accompanying aortic valve disease (aortic stenosis, aortic insufficiency) or bicuspid aortic valve—category 5	25 (11.0%)
Verification of BAV presence—category 3	29 (14.3%)	Dilated ascending aorta in echocardiography without initial diagnosis of BAV or aortic valve disease—category 4	24 (8.9%)	Assessment of the heart and the aorta before TAVI (transcatheter aortic valve implantation)—category 14	17 (7.5%)
Parametric assessment of aortic stenosis or aortic insufficiency coexisting with BAV—category 8	26 (12.8%)	Parametric assessment of acquired aortic valve disease (aortic stenosis, aortic insufficiency) without an initial diagnosis of BAV—category 7	15 (5.5%)	Parametric assessment of acquired aortic valve disease (aortic stenosis, aortic insufficiency) without an initial diagnosis of BAV—category 7	15 (6.6%)
Parametric assessment of acquired aortic valve disease (aortic stenosis, aortic insufficiency) without an initial diagnosis of BAV —category 7	23 (11.3%)	Verification of BAV presence—category 3	15 (5.5%)	Dilated ascending aorta in echocardiography without an initial diagnosis of BAV or aortic valve disease—category 4	13 (5.8%)
Females with BAV in ECG-gated cardiac CT (n = 227)					
≤40 years (n = 54)	n (%)	41–60 years (n = 85)	n (%)	>60 years (n = 88)	n (%)
suspicion or postoperative assessment of congenital cardiovascular defects excluding BAV—category 2	18 (33.3%)	Suspected coronary artery disease—category 1	46 (54.1%)	Suspected coronary artery disease—category 1	43 (48.9%)
verification of BAV presence—category 3	8 (14.8%)	Dilated ascending aorta in echocardiography without an initial diagnosis of BAV or aortic valve disease—category 4	8 (9.4%)	Assessment of the heart and the aorta before TAVI (transcatheter aortic valve implantation)—category 14	11 (12.5%)
dilated ascending aorta in echocardiography with accompanying aortic valve disease (aortic stenosis, aortic insufficiency) or bicuspid aortic valve—category 5	7 (13.0%)	Dilated ascending aorta in echocardiography with accompanying aortic valve disease (aortic stenosis, aortic insufficiency) or bicuspid aortic valve—category 5	8 (9.4%)	Parametric assessment of acquired aortic valve disease (aortic stenosis, aortic insufficiency) without an initial diagnosis of BAV—category 7	7 (8.0%)
Males with BAV in ECG-gated cardiac CT (n = 473)					
≤40 years (n = 149)	n (%)	41–60 years (n = 186)	n (%)	>60 years (n = 138)	n (%)
suspicion or postoperative assessment of congenital cardiovascular defects excluding BAV—category 2	31 (20.8%)	Suspected coronary artery disease—category 1	67 (36.0%)	Suspected coronary artery disease—category 1	56 (40.6%)
dilated ascending aorta in echocardiography with accompanying aortic valve disease (aortic stenosis, aortic insufficiency) or bicuspid aortic valve—category 5	25 (16.8%)	Dilated ascending aorta in echocardiography with accompanying aortic valve disease (aortic stenosis, aortic insufficiency) or bicuspid aortic valve—category 5	40 (21.5%)	Dilated ascending aorta in echocardiography with accompanying aortic valve disease (aortic stenosis, aortic insufficiency) or bicuspid aortic valve—category 5	20 (14.5%)
parametric assessment of aortic stenosis or aortic insufficiency coexisting with BAV—category 8	22 (14.8%)	Dilated ascending aorta in echocardiography without an initial diagnosis of BAV or aortic valve disease—category 4	16 (8.6%)	Dilated ascending aorta in echocardiography without an initial diagnosis of BAV or aortic valve disease—category 4	10 (7.2%)

**Table 4:** Cardiovascular abnormalities coexisting with a bicuspid aortic valve diagnosed with ECG-gated cardiac CT, n = 700. * Others: quadricuspid pulmonary valve (n = 1), right aortic arch (n = 1), atrioventricular septal defect (n = 1). ** Due to the different scan extent in some of the patients, the branches diverging from the aortic arch could not be assessed, therefore the prevalence (%) of some cardiovascular abnormalities in the group n = 700 is not indicated. *** Some subjects had more than one cardiovascular abnormality, therefore the percentage of each group or subgroup of cardiovascular abnormalities in the group n = 700 could not be indicated, only the number of abnormalities.

Cardiovascular Abnormalities Accompanying BAV in CCTA					
Group I. Cardiovascular defects	n (%)	Group II. Coronary artery anomalies	n (%)	Group III. Other cardiovascular abnormalities	n (%)
Ventricular septal defect (VSD)	30 (4.3%)	(1) Anomalies of origin	108 ***	Patent foramen ovale (PFO)	105 (15.0%)
Coarctation of the aorta	25 (3.6%)	High take-off	45 (6.4%)	Atrial septal aneurysm	52 (7.4%)
Atrial septal defect (ASD)	18 (2.6%)	Paracommissural orifice	31 (4.4%)	Accessory left atrial appendage	35 (5.0%)
Partial absence of the pericardium	10 (1.4%)	Absence of left main coronary artery (LMCA)	19 (2.7%)	Bachmann bundle leak	27 (3.9%)
Hypoplastic aortic arch	8 (1.1%)	Origin of coronary artery or branch from opposite sinus	9 (1.3%)	Left ventricular diverticulum	26 (3.7%)
Patent ductus arteriosus (PDA)	7 (1.0%)	Origin of circumflex artery (LCx) from right coronary artery (RCA)	4 (0.6%)	Arteria lusoria	17 (2.4%)
Partial anomalous pulmonary venous return (PAPVR)	7 (1.0%)	(2) Anomalies of termination	3 ***	Common origin of brachiocephalic and left common carotid arteries	13 **
Persistent left superior vena cava	5 (0.7%)	Coronary arteriovenous fistula	3(0.4%)	Left common carotid artery arising from the brachiocephalic trunk	9 **
Transposition of great arteries (TGA)	2 (0.3%)				
Others *	3 (0.4%)				
Total number of cardiovascular defects	115 ***	Total number of coronary artery anomalies	111 ***	Total number of other cardiovascular abnormalities	284 ***

**Table 5:** Gender-specific characteristics of defects coexisting with a bicuspid aortic valve on cardiac CT. * When examining differences between males and females in the incidence of clinically significant cardiovascular abnormalities, a quasi-significant result was obtained for VSD (*p* = 0.059).

Characteristics of Study Group		Males		Females		
Number of patients with clinically relevant cardiovascular abnormalities, n (%)			111 (23.5%)		58 (25.6%)	
Number of clinically relevant cardiovascular abnormalities			143		83	
Number of defects/person in patients with clinically relevant cardiovascular abnormalities			1.29		1.43	
Number of defects/person in the whole group			0.30		0.37	
% of patients with left-dominant coronary circulation in the group with clinically relevant cardiovascular abnormalities			17.1%		25.9%	
% of patients with left-dominant coronary circulation in the whole group			14.4%		15.0%	
Most common clinically relevant cardiovascular abnormalities, n (%)		High take-off	34 (7.2%)	Paracommissural orifice	17 (7.5%)	
		VSD	25 (5.3%)	Coarctation of the aorta	11 (4.9%)	
		Absence of LMCA	15 (3.2%)	High take-off	11 (4.9%)	
		Coarctation of the aorta	14 (3.0%)	ASD	8 (3.5%)	
		Paracommissural orifice	14 (3.0%)	PAPVR	7 (3.1%)	
Defects with different prevalences in females and males	Paracommissural orifice, n (%)		14 (3.0%)		17 (7.5%)	*p* = 0.010
	PAPVR, n (%)		0 (0.0%)		7 (3.1%)	*p* < 0.001
	VSD *, n (%)		25 (5.6%)		5 (2.2%)	*p* = 0.059

**Table 6:** Defects co-occurring with a bicuspid aortic valve on ECG-gated cardiac CT, divided by BAV types and subtypes.

Characteristics of the Study Group	Type 0 (n = 42)		Type 1 (n = 601)		Type 2 (n = 57)	
Number of patients with clinically relevant cardiovascular abnormalities, n (%)		15 (35.7%)		143 (23.8%)		11 (19.3%)
Number of clinically relevant cardiovascular abnormalities		22		187		17
Number of defects/person in patients with clinically relevant cardiovascular abnormalities		1.47		1.30		1.54
Number of defects/person in the whole group		0.52		0.31		0.30
% of patients with left-dominant coronary circulation in the group with clinically relevant cardiovascular abnormalities		40.0%		17.5%		27.3%
% of patients with left-dominant coronary circulation in the whole group		21.4%		13.6%		19.3%
Most common clinically relevant cardiovascular abnormalities, n (%)	High take-off	5 (11.9%)	High take-off	33 (5.5%)	High take-off	7 (13.5%)
	Coarctation of the aorta	4 (9.5%)	Paracommissural orifice	29 (4.8%)	VSD	2 (3.8%)
	VSD	2 (4.8%)	VSD	25 (4.2%)	PDA	2 (3.8%)
	ASD	2 (4.8%)	Coarctation of the aorta	21 (3.5%)		
	Absence of LMCA	2 (4.8%)	Absence of LMCA	16 (2.7%)		
Most common clinically relevant cardiovascular abnormalities in particular BAV subtypes, n (%)	Type 0 lateral (n = 18)		Type 1 R-L (n = 501)			
	High take-off	5 (27.8%)	High take-off	27 (5.4%)		
	Coarctation of the aorta	3 (16.7%)	Paracommissural orifice	25 (5.0%)		
	ASD	2 (11.1%)	VSD	22 (4.4%)		
	Type 0 A-P (n = 24)		Type 1 R-N (n = 87)			
	VSD Absence of LMCA	2 (8.3%) 2 (8.3%)	High take-off	6 (6.9%)		
			Coarctation of the aorta	5 (5.7%)		
			Paracommissural orifice	4 (4.6%)		
			Type 1 L-N (n = 13)			
			Only three defects:			
			ASD	1 (7.7%)		
			VSD	1 (7.7%)		
			TGA	1 (7.7%)		

**Table 7:** Most common cardiovascular abnormalities coexisting with a bicuspid aortic valve on ECG-gated cardiac CT by age group and gender of subjects.

Characteristics of the Study Group	≤40 Years (n = 203)		41–60 Years (n = 271)		>60 Years (n = 226)	
Number of patients with clinically relevant cardiovascular abnormalities, n (%)		60 (29.6%)		67 (24.7%)		42 (18.6%)
Number of clinically relevant cardiovascular abnormalities		98		78		50
Number of defects/person in patients with clinically relevant cardiovascular abnormalities		1.63		1.16		1.19
Number of defects/person in the whole group		0.48		0.29		0.22
% of patients with left-dominant coronary circulation in the group with clinically relevant cardiovascular abnormalities		23.3%		17.9%		19.0%
% of patients with left-dominant coronary circulation in the whole group		17.7%		15.1%		11.1%
the most common clinically relevant cardiovascular abnormalities, n (%)	High take-off	21 (10.3%)	Absence of LMCA	14 (5.2%)	Paracommissural orifice	11 (4.9%)
	Coarctation of the aorta	18 (8.9%)	High take-off	14 (5.2%)	High take-off	10 (4.4%)
	VSD	13 (6.4%)	Paracommissural orifice	11 (4.1%)	VSD	7 (3.1%)
	Paracommissural orifice	9 (4.4%)	VSD	10 (3.7%)	ASD	6 (2.7%)
	ASD	7 (3.4%)	Coarctation of the aorta	7 (2.6%)	Origin of coronary artery or branch from opposite sinus	5 (2.2%)
	Females ≤ 40 years (n = 54)		Females 41–60 years (n = 85)		Females > 60 years (n = 88)	
the most common clinically relevant cardiovascular abnormalities, n (%)	High take-off	17 (11.4%)	Paracommissural orifice	5 (5.9%)	Paracommissural orifice	5 (5.7%)
	VSD	11 (7.4%)	High take-off	3 (3.5%)	High take-off	3 (3.4%)
	Coarctation of the aorta	9 (6.0%)	VSD	2 (2.4%)	Origin of coronary artery or branch from opposite sinus	3 (3.4%)
number of defects/person ratio		0.69		0.28		0.24
% of patients with left-dominant coronary circulation		16.7%		11.8%		17.0%
	Males ≤ 40 years (n = 149)		Males 41–60 years (n = 186)		Males > 60 years (n = 138)	
Most common clinically relevant cardiovascular abnormalities, n (%)	High take-off	17 (11.4%)	Absence of LMCA	12 (6.5%)	High take-off	7 (5.1%)
	VSD	11 (7.4%)	High take-off	10 (5.4%)	Paracommissural orifice	6 (4.3%)
	Coarctation of the aorta	9 (6.0%)	VSD	8 (4.3%)	VSD	6 (4.3%)
Number of defects/person		0.41		0.29		0.21
% of patients with left-dominant coronary circulation		18.1%		16.7%		7.2%

**Table 8:** The differences in coronary artery stenosis severity between subjects with a bicuspid aortic valve (BAV) and those with a tricuspid aortic valve (TAV) compared to the control group.

Coronary Artery Stenosis Severity (0–4)	BAV (n = 700)	TAV (n = 100)	*p*-Value
Category 0, n (%)	221 (31.6%)	42 (42.0%)	0.037
Category 1, n (%)	346 (49.4%)	46 (46.0%)	NS
Category 2, n (%)	12 (1.7%)	2 (2.0%)	NS
Category 3, n (%)	89 (4.8%)	8 (8.0%)	NS
Category 4, n (%)	32 (12.7%)	2 (2.0%)	NS
Stenosis > 50% (≥category 2 stenosis severity), n (%)	143 (20.4%)	12 (12.0%)	0.046

**Table 9:** Correlation coefficients of coronary artery calcium (CAC) and age in patients with different types of BAVs on ECG-gated CT determined using Spearman’s test.

	Type 0	Type 1 R-L	Type 1 R-N + 1 L-N	Type 2
Age	0.59	0.58	0.52	0.46

**Table 10:** Distribution of CAC scores by the type of bicuspid aortic valve on cardiac CT. Values for type 1 are shown both with and without division into subtypes 1 R-L and 1 R-N + 1 L-N.

BAV Type	Type 0 (n = 42)	Type 1 R-L (n = 501)	Type 1 R-N + 1 L-N (n = 100)	Type 1 Total (n = 601)	Type 2 (n = 57)
Age (years), mean ± SD	46.6 ± 20.0	51.4 ± 18.6	49.0 ± 19.3	51.0 ± 18.7	29.8 ± 15.5
Males, %	71.4%	68.3%	60.0%	66.9%	71.9%
Agatston score (U), mean ± SD	193.4 ± 590.2	158.8 ± 508.9	212.7 ± 435.7	167.8 ± 497.5	4.8 ± 17.7
Classification of Agatston score					
0 U	27 (64.3%)	276 (55.1%)	59 (59.0%)	335 (55.7%)	49 (86.0%)
P1 (1–100 U), n (%)	6 (14.3%)	114 (22.8%)	11 (11.0%)	125 (20.8%)	7 (12.3%)
P2 (101–300 U), n (%)	4 (9.5%)	44 (8.8%)	8 (8.0%)	52 (8.7%)	1 (1.8%)
P3 (301–999 U), n (%)	1 (2.4%)	50 (10.0%)	16 (16.0%)	66 (11.0%)	0 (0.0%)
P4 (≥1000 U), n (%)	4 (9.5%)	17 (3.4%)	6 (6.0%)	23 (3.8%)	0 (0.0%)
Agatston score percentiles (U)					
25th	0	0	0	0	0
50th	0	0	0	0	0
75th	32.5	84.0	224.5	87.0	0
90th	294.4	425.0	717.4	510.0	14.2
95th	1230.6	805.0	1115.7	846.0	19.2

**Table 11:** Distribution of CAC score according to age and gender in patients with BAV on cardiac CT. Values for type 1 are presented both with and without division into subtypes 1 R-L and 1 R-N + 1 L-N.

Age Group	≤40 Years			41–60 Years			>60 Years		
Characteristics of age group	Females (n = 54)	Males (n = 149)	Total (n = 203)	Females (n = 85)	Males (n = 186)	Total (n = 271)	Females (n = 88)	Males (n = 138)	Total (n = 226)
Agatston score (U), mean ± SD	0.0 ± 0.0	4.2 ± 45.7	3.1 ± 39.1	28.7 ± 116.9	127.3 ± 295.7	96.4 ± 257.4	214.9 ± 446.7	460.7 ± 895.5	365.0 ± 761.5
Classification of Agatston score									
0 U, n (%)	54 (100.0%)	143 (96.0%)	197 (97.0%)	58(68.2%)	93 (50.0%)	151(55.7%)	30 (34.1%)	33 (23.9%)	63 (27.9%)
P1 (1–100 U), n (%)	0 (0.0%)	5 (3.6%)	5 (2.5%)	21 (24.7%)	49 (26.3%)	70 (25.8%)	27 (30.7%)	36 (26.1%)	63 (27.9%)
P2 (101–300 U), n (%)	0 (0.0%)	0 (0.0%)	0 (0.0%)	5 (5.9%)	19 (10.2%)	24 (8.9%)	14 (15.9%)	19 (13.8%)	33 (14.6%)
P3 (301–999 U), n (%)	0 (0.0%)	1 (0.7%)	1 (0.5%)	0 (0.0%)	21 (11.3%)	21 (7.7%)	12 (13.6%)	33 (23.9%)	45 (19.9%)
P4 (≥1000 U), n (%)	0 (0.0%)	0 (0.0%)	0 (0.0%)	1 (1.2%)	4 (2.2%)	5 (1.8%)	5 (5.7%)	17 (12.3%)	22 (9.7%)
Agatston score percentiles (U)									
25th	0	0	0	0	0	0	0	0	0
50th	0	0	0	0	0	0	82.5	82.5	78
75th	0	0	0	78	61	44.5	547.3	508.5	408.5
90th	0	0	0	378	388.5	274	1116.8	1133.4	952.5
95th	0	0	0	529.8	773.5	666	1581.2	1805.6	1525.8

**Table 12:** Coronary artery stenosis severity in subjects with different types of BAVs on cardiac CT according to the Sievers–Schmidtke classification, n = 700. Type 1 was divided into subtypes 1 R-L and 1 R-N + 1 L-N.

Coronary Artery Stenosis Severity (0–4)	Type 0 (n = 42)	Type 1 R-L (n = 501)	Type 1 R-N + 1 L-N (n = 100)	Type 2 (n = 57)	*p*-Value
Median (IQR)	1 (0–1)	1 (0–1)	1 (0–3)	0 (0–1)	<0.001
Category 0, n (%)	15 (35.7%)	133 (26.5%)	34 (34.0%)	39 (68.4%)	<0.001
Category 1, n (%)	20 (47.6%)	276 (55.1%)	33 (33.0%)	17 (29.8%)	<0.001
Category 2, n (%)	3 (7.1%)	8 (1.6%)	1 (1.0%)	0 (0%)	<0.001
Category 3, n (%)	2 (4.8%)	66 (13.2%)	21 (21.0%)	0 (0%)	<0.001
Category 4, n (%)	2 (4.8%)	18 (3.6%)	11 (11.0%)	1 (1.8%)	<0.001
Stenosis > 50% (≥category 2 stenosis severity), n (%)	7 (16.7%)	92 (18.4%)	33 (33.0%)	1 (1.8%)	<0.001

**Table 13:** Univariate analysis of demographic and cardiac computed-tomography-based parameters (calcium score).

	OR [95%CI]	*p*-Value
Gender (0—females; 1—males)	1.56 [1.01, 2.39]	0.044
Age (continuous)	1.07 [1.05, 1.09]	<0.001
Type of BAV (0—other types of BAV; 1—type 1 R-N + 1 L-N)	2.46 [1.54, 3,94]	<0.001
Calcium score (continuous)	1.007 [1.006, 1.008]	<0.001

**Table 14:** Results of logistic regression analysis.

		Estimate	SE	*t*-Test	*p*-Value	Odds Ratio	−95% CI	+95% CI
b0	Intercept Log. reg.	−4.926	0.586	70.48	<0.001	0.007	0.002	0.023
b1	Age	0.042	0.010	19.40	<0.001	1.043	1.024	1.063
b2	BAV type	0.997	0.333	8.97	0.003	2.709	1.411	5.201
b3	Calcium score	0.006	0.001	71.46	<0.001	1.006	1.004	1.007

## Data Availability

The original contributions presented in the study are included in the article, further inquiries can be directed to the corresponding authors.
